# Unveiling a Hidden Event in Fluorescence Correlative Microscopy by AFM Nanomechanical Analysis

**DOI:** 10.3389/fmolb.2021.669361

**Published:** 2021-05-06

**Authors:** Massimiliano Galluzzi, Bokai Zhang, Han Zhang, Lingzhi Wang, Yuan Lin, Xue-Feng Yu, Zhiqin Chu, Jiangyu Li

**Affiliations:** ^1^ Materials Interfaces Center, Shenzhen Institutes of Advanced Technology, Chinese Academy of Sciences, Shenzhen, China; ^2^ Shenzhen Key Laboratory of Nanobiomechanics, Shenzhen Institutes of Advanced Technology, Chinese Academy of Sciences, Shenzhen, China; ^3^ DGene (Dongjin Big Health (Shenzhen)) Co., Ltd., Shenzhen, China; ^4^ BenHealth Biopharmaceutical (Shenzhen) Co., Ltd., Shenzhen, China; ^5^ State Key Laboratory of Traction Power, Southwest Jiaotong Univerisity, Chengdu, China; ^6^ Department of Electrical and Electronic Engineering, The University of Hong Kong, Hong Kong; ^7^ Department of Mechanical Engineering, The University of Hong Kong, Hong Kong; ^8^ Advanced Biomedical Instrumentation Centre, Shatin, Hong Kong; ^9^ Department of Electrical and Electronic Engineering, Joint Appointment with School of Biomedical Sciences, The University of Hong Kong, Hong Kong; ^10^ Department of Materials Science and Engineering, Southern University of Science and Technology, Shenzhen, China

**Keywords:** atomic force microscopy, correlative fluorescence microscopy, biomembrane, fluorophore, hybrid phospholipids

## Abstract

Fluorescent imaging combined with atomic force microscopy (AFM), namely AFM-fluorescence correlative microscopy, is a popular technology in life science. However, the influence of involved fluorophores on obtained mechanical information is normally underestimated, and such subtle changes are still challenging to detect. Herein, we combined AFM with laser light excitation to perform a mechanical quantitative analysis of a model membrane system labeled with a commonly used fluorophore. Mechanical quantification was additionally validated by finite element simulations. Upon staining, we noticed fluorophores forming a diffuse weakly organized overlayer on phospholipid supported membrane, easily detected by AFM mechanics. The laser was found to cause a degradation of mechanical stability of the membrane synergically with presence of fluorophore. In particular, a 30 min laser irradiation, with intensity similar to that in typical confocal scanning microscopy experiment, was found to result in a ∼40% decrease in the breakthrough force of the stained phospholipid bilayer along with a ∼30% reduction in its apparent elastic modulus. The findings highlight the significance of analytical power provided by AFM, which will allow us to “see” the “unseen” in correlative microscopy, as well as the necessity to consider photothermal effects when using fluorescent dyes to investigate, for example, the deformability and permeability of phospholipid membranes.

## Introduction

Fluorescence microscopy is one of the most often used imaging techniques in biology and material science (Lichtman and Conchello, 2005; Shashkova and Leake, 2017), and this is dictated by efficient staining agents with ability to bind on target while maintaining fluorescence. The high-specificity of fluorophores is relative to the interaction force between those label agents and target biomolecules. While process of measurement requires to minimize the perturbation of system under investigation, a strong (for example covalent (Luitz et al., 2017)) label-target interaction can produce an opposite effect. Complementary investigations are essential to unveil the influence of fluorescent tags, as well as to understand the targeting quality and uniformity.

A peculiar way enabled by atomic force microscopy (AFM), is to investigate the mechanical properties in biological system through quantitatively measuring viscoelastic modifications (Nautiyal et al., 2018; Garcia, 2020; Zhou et al., 2021). Considering the versatility of AFM in biology, it is significant to integrate such platform with optics, the so-called correlative microscopy (Zhou et al., 2021). With such a combination, the mechanical quantities and biochemical events can be simultaneously investigated with high spatial-temporal resolution. This will provide new insights into biological processes, but previous studies also suggested the used fluorophores could have huge impact on the targeted biomolecules during visualization (Luitz et al., 2017; Cosentino et al., 2019). Highly desirable now is to understand the underlying cross-talk between the two techniques, especially in a quantitative manner.

For our study we selected one of the most studied bio-systems, represented by phospholipid bilayers as a model system mimicking real cell membrane (Eeman and Deleu, 2010; Redondo-Morata et al., 2020). Furthermore, the modeled biomembranes can be flexibly reconstructed to facilitate surface techniques for investigation of mechanical properties such as AFM (Stetter et al., 2016; Müller et al., 2020; Redondo-Morata et al., 2020), and molecular dynamic simulations (Hofsäß et al., 2003; Leeb and Maibaum, 2018). This is significant since the biomembrane is the most critical interface between the living organisms and the external environment, while mechanical properties are correlated with its stability (Alessandrini and Facci, 2012; Beedle et al., 2015; Balleza et al., 2019; Mescola et al., 2020).

In current work, we perform an AFM-fluorescence correlative microscopy study on a model system consisting of biomembrane (DOPC bilayer) interacting with a commonly used hydrophobic molecule, Nile red (Ira and Krishnamoorthy, 1998; Jain and Das, 2006; Kurniasih et al., 2015; Levitt et al., 2015; Halder et al., 2018), having high efficacy staining intracellular neutral lipid droplets and phospholipid cell membranes. Very recently, Zhanghao et al. (Zhanghao et al., 2020) used Nile red to perform single-cell super-resolution lipidomic resolving the membrane morphology, polarity, and phase of cell membrane and subcellular organelles. Lipid order in outer leaflet of bacterial membrane was investigated with high-sensitivity fluorescent probe based on Nile red (Bogdanov et al., 2020). Therefore, the investigation of mechanical properties of membrane in physiological conditions will be essential to determine a cross-talk induced by fluorescent optical microscopy. Our obtained results are well-validated by direct comparison of AFM single force spectra with finite element simulations (FEM) based on AFM topography. We compare the cases of model system with or without laser irradiation, and show different degrees of mechanical modifications. Moreover, we used our routine on a hybrid system composed by DOPC + Nile red before aqueous micellization, again highlighting the synergistic effect of laser and fluorophore in modifying fluidity and stability of lipid-based membranes.

## Materials and Methods

### Phospholipid Bilayer Preparation and Red Nile

The phospholipid molecules (dioleoyl phosphatidylcholine (DOPC) from Sigma-Aldrich, St. Louis, MO) were initially dissolved in a chloroform solution at a concentration of 10 mg/ml. The dried lipids (0.1 mg) were rehydrated in PBS (phosphate buffer saline) maintaining 0.5 mg/ml concentration, the final solution increases turbidity due to formation of micelles. The ionic strength of PBS is necessary to shield effectively the electrostatic repulsive interaction between the negatively charged mica surface and phospholipids heads (Reviakine and Brisson, 2000; Richter and Brisson, 2005; Indrieri et al., 2008). The lipids solution is vortexed and sonicated for 10 min. The effect of sonication was to break bigger multi-layered vesicles, favoring the formation of smaller single-layered vesicles. After sonication, the solution containing the lipids is deposited on a freshly cleaved mica surface in the amount of 40 μl with 2 min incubation time. The deposition protocol proceeds with the elimination of deposition buffer by spin coating at 5000 rpm for 2 min. We found that spin coating was the best methodology to control the quantity of single bilayers on mica substrate, achieving repeatability. Finally, the sample was rehydrated in PBS to perform AFM measurement in fluid environment using the imaging buffer. In this work, PBS was utilized to mimic closer physiological conditions. Importantly, the supported bilayers can be stabilized by the higher ionic strength on mica (therefore it is easier to find bilayers during microscopy). Moreover, the electrostatic interaction is shielded by ionic strength. The “unshielded” electrostatic interaction in liquid (described by DLVO theory) is increasing uncertainty during contact point determination. The overall procedure was tested several times in order to ensure repeatability.

Nile Red (N1142, Invitrogen, Carlsbad, CA) was firstly dissolved in DMSO at a concentration of 10 mg/ml, then the solution was further diluted to a final concentration of 2.5 μg/ml in PBS, which was applied as the imaging buffer. CellTracker Deep Red (C34565, Thermo Fisher Scientific, Waltham, MA) and HOECHST (33,342, Thermo Fisher Scientific) were dissolved in PBS with concentrations of 25 μg/ml and 2.5 μg/ml, respectively.

### Laser Installation and Alignment

To simulate the confocal microscope environment to the maximum extent, we installed the 532 nm laser (green) to the optical microscopy (Eclipse Ti2, Nikon, Tokyo, Japan) as the light source for the excitation of Nile Red and CellTracker Deep Red, while 480 nm laser (blue) was used for HOECHST. In brief, a front-surface mirror was placed on the stage of the microscopy, the installed laser was adjusted to focus onto the reflective surface using the 40X objective lanes. Then filter settings were selected to allow some of the reference laser to be bounced back through the microscopy optical train and out through the external coupling port. After alignment, the laser power intensity is adjusted by the controller. In our case, 10.1 mW power intensity was set to obtain a final output power intensity of 35.4 mW/μm^2^. More details on this calculation are collected in Supporting Information, Supplementary Figure SI1.

### AFM Force Spectroscopy

AFM force spectroscopy experiments were performed with MFP 3DBio (Asylum Research, Goleta, CA) mounting short-wide SNL probes (Bruker, Billerica, MA) with nominal elastic constant k = 0.5 N/m, nominal radius R = 10 nm, allowing to cover force interaction range from 10–10^4^ pN. The elastic constant of cantilever was calibrated by thermal tuning with built-in procedure in air and in water leading to consistent results of elastic constant as k = 0.42 N/m. We used the environmental control unit to keep the temperature constant at 30°C, slightly higher than room temperature to allow the system to re-equilibrate in case of thermal modification induced by AFM operation and laser application.

During AFM force spectroscopy, the probe approaches vertically the phospholipid bilayer recording the interaction force in function of probe-surface position. The first visible nanomechanical parameter is related to the quantitative determination of the breakthrough event, i.e. the force threshold indicative of AFM probe penetrating the lipid layer, also confirming its existence (Butt et al., 2005; Redondo-Morata et al., 2020). Raw morphology from integrated software is representative of mica substrate, neglecting layers breaking during force curves. For each sample, a series of force volume mapping at 64 × 64 resolution was performed insisting on the same area but using different conditions (dye injection and/or laser application) in different macroscopic positions to improve the statistical reliability of the experiments.

### Data Analysis

Data analysis was performed in MATLAB (The MathWorks, Natick, MA) environment with custom routine functions. Analysis is firstly focused in converting raw force curves from Deflection Voltage (V) vs. Piezo Travel Distance (nm) into Force (nN) vs. Tip-substrate separation (nm)(Butt et al., 2005). To this purpose, optical lever sensitivity Zsens (nm/V) is calculated from contact part when probe reached the substrate after breaking through the layer, while spring constant is used to finally convert in Force.

**FIGURE 1:**
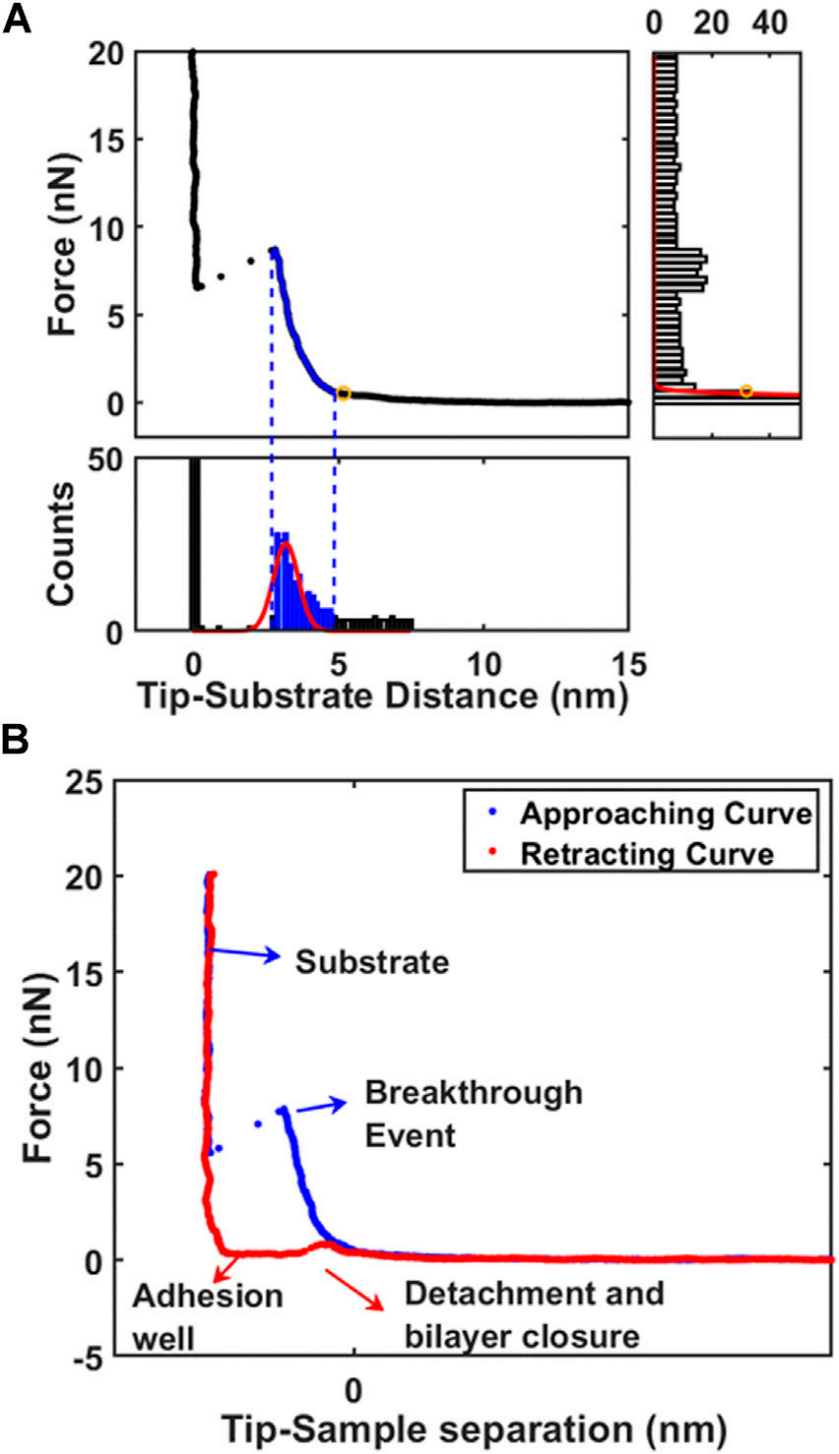
The analysis of supported phospholipid bilayers. **(A)** Working principle of algorithm to identify automatically the contact point on first layer by decomposition of force curve (FC) with X-Y histograms. **(B)** Typical force curve (approaching and retracting) acquired on phospholipid bilayer highlighting important positions.

After conversion, the tip-sample contact point must be determined to consider the morphology of soft biomembranes (Galluzzi et al., 2018). As first approach, the force axis is binned resulting in a well-defined peaked histogram indicating non-contact region (right part of Figure 1A). To avoid considering electrostatic interactions, the low-force morphology is reconstructed individuating contact point above 0.3 nN. The second approach is focused in finding the layer near the substrate by binning the distance axis. While the peak correspondent to zero distance is representing the substrate, the second peak represents the nearest layer (bottom part of Figure 1A). Gaussian fitting is employed to find automatically the peaks for all force curves in force volume. The second approach is particularly powerful to exclude long-range electrostatic interactions or loosely bounded contaminants on surface of phospholipid bilayer. Using the peak correspondent to first layer facing the substrate, the contact point and breakthrough force can be retrieved easily as left and right extremes of Gaussian fitting. After contact with surface, AFM probe starts to compress the bilayer until the indentation reaches circa 2.5 nm, at which point the elastic energy of the deformed bilayer overcomes the energetic cost of forming a hole and the tip penetrates the bilayer reaching the substrate in a breakthrough event (Figure 1B). (Garcia-Manyes et al., 2005; Das et al., 2010; Garcia-Manyes and Sanz, 2010) The breakthrough force is a parameter representing the mechanical stability to vertical compressive loads. After reaching the maximum force setpoint, the tip reverses the motion and retracts from surface allowing to measure the force necessary to detach from surface and close the hole in phospholipid bilayer (red curve in Figure 1B).

The first layer topography map is obtained from difference between contact point and substrate, so that thickness map (from h parameter) is equivalent to standard morphology. The contact part of force curve between contact point and F_break_ is then used for mechanical analysis. Considering the typical probe radius can be approximated by sphere which radius is larger than indentation length (usually 2 nm for our system), we consider standard Hertz model with the correction for finite thickness to describe a system composed by a thin elastic layer supported on solid substrate (Garcia-Manyes and Sanz, 2010; Garcia-Manyes et al., 2005; Das et al., 2010). For the elastic contribution, we implement the theoretical model represented in second term of Eq 1: (Dimitriadis et al., 2002; Stetter et al., 2016)

$$F=\frac{4}{3}\frac{E\sqrt{R}}{\left(1-\nu^{2}\right)}\delta^{3/2}\left[1+1.133\chi_{S}+1.283\chi_{S}^{2}+0.769\chi_{S}^{3}+0.0975\chi_{S}^{4}\right];\;\chi_{S}=\frac{\sqrt{R\delta }}{h}$$ (1)

Where F is the applied force, δ the indentation, ν the Poisson’s ratio, E the effective Young’s Modulus of the lipid bilayer, and R the radius of the spherical probe and the adimensional parameter $\chi_{S}=\sqrt{R\delta /h}$. Poisson ratio for lipid bilayers is close to 0.5, with relative deviations about 3%. (Terzi et al., 2019) For a single measurement, the error σ associated with the mean thickness, F_break_ or Young’s modulus of the membrane is calculated taking into account the propagated uncertainty in calibration procedure and the width of histogram distribution correspondent to a single measurement. We highlight that quantification of mechanical parameters is difficult or hardly reproducible, because the geometry of apex of sharp probe is unknown or irregular, moreover the choice of probe geometry and correspondent model is causing largest differences in the interpretation of the data. (Eid et al., 2021) Here we simplify using a probe with apex approximated as a sphere with nominal radius R = 10 nm, moreover we managed to use the very same probe for all experiments and performing comparisons relative to control specimen. Between different experiments AFM probe was cleaned by gentle flux of ethanol.

### FEM Simulations

A 2D-axisymmetric numerical model was developed to study indentation on a supported phospholipid bilayer with a spherical indenter, mimicking AFM experiments as closely as possible. The total size of bilayer was 1 um in lateral dimension (radius) and 5 nm layer supported on 500 nm thick mica (can be considered as thin slab supported on semi-infinite substrate). The radius of the spherical indenter was set as in AFM experiments, R = 10 nm. During deformation, the left boundary, representing the symmetry axis, was allowed to move only vertically, while the bottom boundary was constrained to move horizontally. The right and top boundaries were not constrained, except when indenter is contacting the top objects (surface or inclusions): the contact area was restricted to follow the indenter contour ensuring hard contact between the indenter and the sample. Material properties for substrate (mica), and indenter (silicon) are required as input parameters in the modeling and set as *E*_substrate_ = 3.3 GPa, *ν*_substrate_ = 0.34, *E*_Si_ = 160 GPa, *ν*_Si_ = 0.22. These hard materials have Young’s moduli several orders of magnitude larger (GPa range) than phospholipid bilayers (usually in the MPa range (Jacquot et al., 2014; Stetter et al., 2016)) in this study, showing negligible deformations. Young’s moduli of phospholipid bilayer were directly measured by AFM indentation experiments. A methodological study, comparing AFM indentation and FEM model was recently published by our group (Tang et al., 2018; Tang et al., 2019).

Stress and deformation fields (example is reproduced in Supplementary Figure SI2) produced by indenter approaching vertically are directly converted in Force (nN) vs. Indentation (nm) to be compared with AFM approaching force curves.

An array of simulation was generally built variating a single unknown quantity around the most probable value guessed from AFM morphology and mechanical analysis (Thickness and corrected Young’s Modulus). FEM model can be designed tuning the slip/friction at the interface between thin layer and substrate: 2 extreme conditions are represented by perfect bound (zero slip) and perfect loose (free slip). Although the deformation pattern at the interface at zero and free slip can be qualitatively similar, only quantitative analysis through AFM comparison can distinguish the proper behavior at the interface.

## Results and Discussion

### The Issues in AFM-combined Fluorescence Correlative Microscopy

**FIGURE 2:**
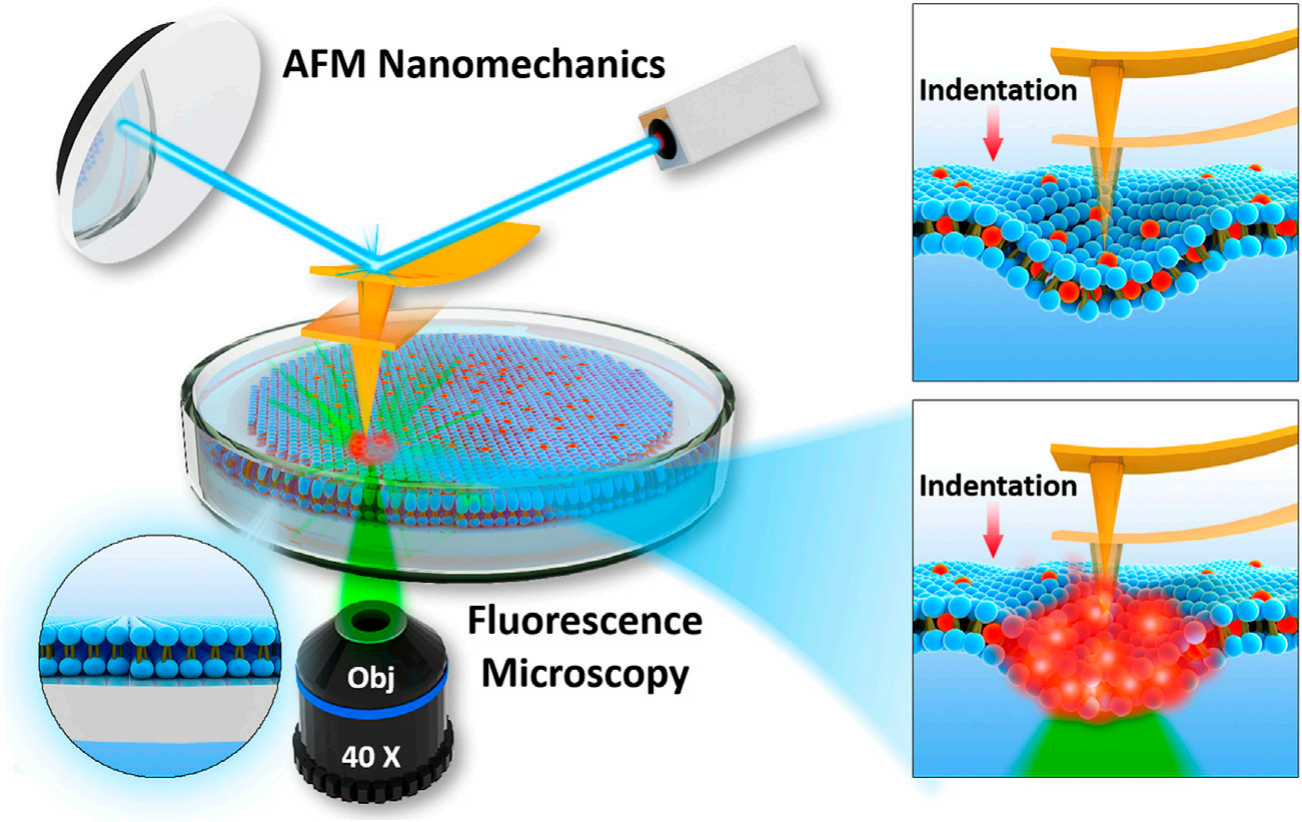
Schematics of integration of AFM with fluorescence microscopy used to determine nanomechanics of phospholipid bilayers interacting with fluorophore and focused laser light.

The commonly integrated fluorescence microscopy, especially the confocal laser scanning microscopy (CLSM), raises the concern of photochemistry effects induced by tightly focused laser beam, particularly in the presence of dye molecules. Whether these photochemical effects can affect the mechanical properties becomes critical, but difficult to detect. Figure 2 represents the schematic of experimental setup used in current study, to perform laser fluorescent microscopy co-localized with atomic force microscopy. Before all the experiments, laser alignment on AFM probes was achieved as explained in Materials and Methods. We ensured that intrinsic noise during force spectroscopy was not modified during laser application.

When exposed in dry air, a progressive bending of the probe was observed presumably due to local heating induced by intensely focused green laser. However, such effect became negligible when the probe was immersed in water with temperature controlled at 30 °C. Also, we noticed that global thermal output remains constant even after 1 h of green (or blue) laser irradiation, showing that excitation laser is not influencing (with 0.1°C resolution) the global temperature of cell dish containing 2 ml PBS.

Standard laser confocal scanning microscopy setup was then adopted where laser spot was focused on surface through microscopy objective, so that local power density of laser was enhanced. In Supplementary Figure SI1 we show the schematic of laser focused application, along with calculation of laser power density. All experiments involving green laser excitation were maintained at the same output power measured directly on sample stage as P = 10.1 mW. Laser beam was focused onto sample surface with a 40X objective lens finally leading to a local power density around 35.4 mW/μm^2^ (see Supporting Information, section: Focusing the laser for more details).

### AFM Mechanical Imaging of Phospholipid Bilayers

**FIGURE 3:**
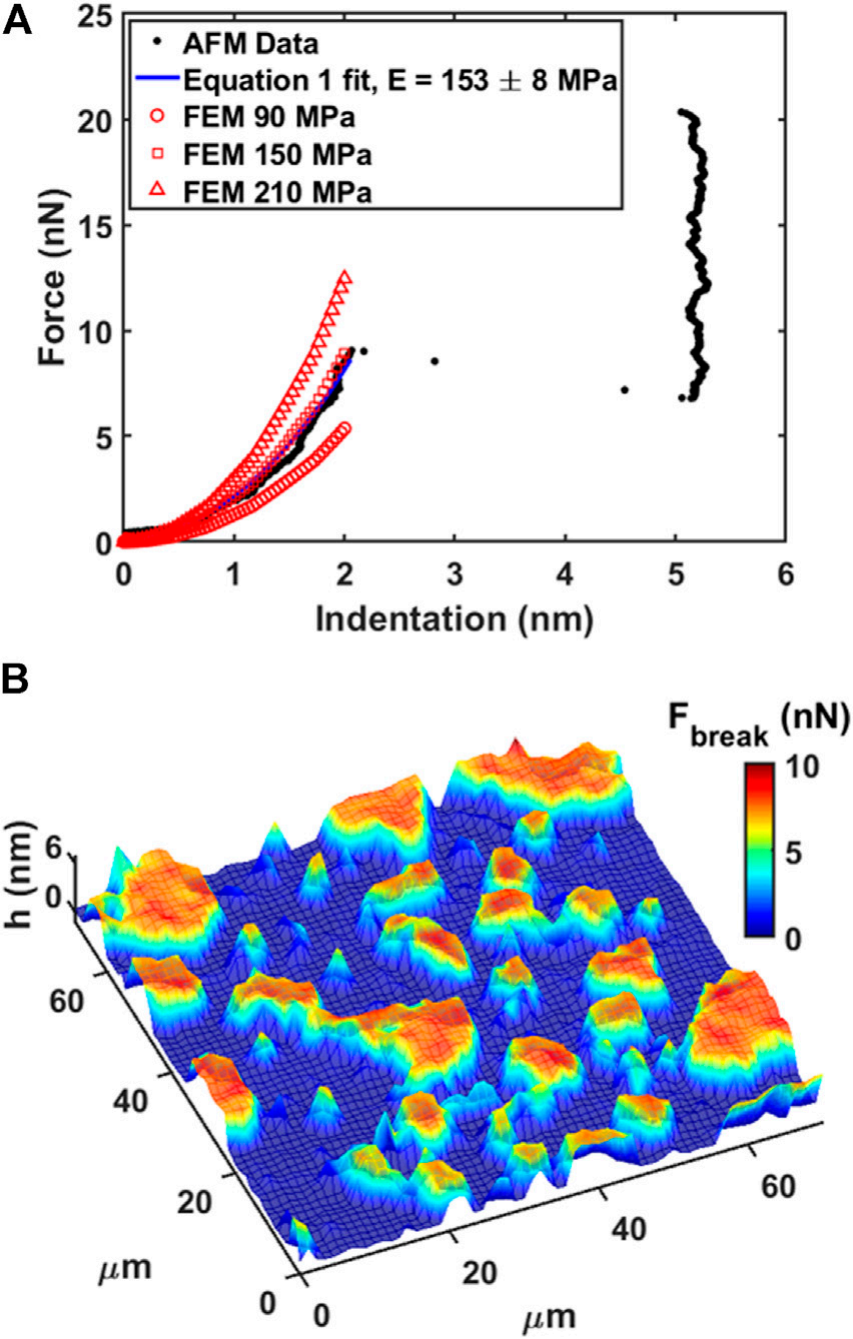
Force spectroscopy and force volume on phospholipid bilayer. **(A)** Force vs. Indentation with fitting of Eq. 1 and FEM simulated curves overlapped. **(B)** 3D Map representation of 64 × 64 force volume grid by overlapping breakthrough force color map on morphology. Low ‘h’ thickness (blue color) represents substrate, while higher ‘h’ thickness (red color) represents the bilayer.

DOPC phospholipid bilayers were deposited on mica and AFM measurements were performed as described in Materials and Methods. As an example, we showed a typical AFM mechanical investigation by indenting a mica substrate partially covered by DOPC bilayers in Figure 3. Between contact point and breakthrough event, the contact part was fitted using Eq. 1 in order to retrieve the Young’s modulus value after finite thickness correction (Dimitriadis et al., 2002). As shown in Figure 3A, the fitting curve follows the experimental data resulting in a final modulus of 153 ± 8 MPa. Following the generic mechanical approach and using simple Hertz model derivation, Young’s modulus results in 406 ± 7 MPa, largely overestimating the modulus due to the hard substrate contribution. In addition, we built an array of FEM simulations (Supplementary Figure SI2) using same thickness of experimental data (5 nm), different Young’s moduli values and free to slip boundary conditions (as expected from fluid-phase DOPC). In Figure 3A the simulated indentation curves are overlapped to data and Eq. 1 fit showing that finite thickness correction is a good approximation and necessary to extract quantitative mechanical properties.

In our experiments, DOPC supported bilayers on mica were investigated by repeating single force spectroscopy events along a regular matrix, forming a Force Volume (FV). This approach is finally leading to property maps and corresponding histograms of distributed values to improve the statistic reliability. As an example, in Figure 3B we show the overlay of breakthrough force map on 3D morphology while highlighting the pixel/force spectroscopy grid. The values obtained during control test analysis (shown in Table 1) were in agreement with values found in literature (Das et al., 2010; Garcia-Manyes and Sanz, 2010; Alessandrini and Facci, 2012).

**TABLE 1:** Summary of experimental setting parameters and obtained mechanical properties. For each experiment we show the percentual variability of selected conditions in comparison with control.

Condition	Thickness (nm)	Breakthrough (nN)	Young’s modulus (MPa)	Adhesion (pN)
Control	4.67 ± 0.53	7.13 ± 0.73	157 ± 22	74 ± 11
Time 3 h	4.48 ± 0.65	7.28 ± 0.98	152 ± 34	47 ± 5
	−4%	2%	−3%	-36%
Control	4.62 ± 0.46	8.67 ± 1.12	169 ± 27	89 ± 11
T = 41°C	4.41 ± 0.34	7.53 ± 0.69	153 ± 22	101 ± 19
	−4%	−15%	−10%	11%
Control	4.85 ± 0.63	7.68 ± 0.64	149 ± 30	30 ± 5
Laser green	4.72 ± 0.54	6.89 ± 0.48	144 ± 31	31 ± 6
	−3%	−12%	−3%	3%
Control	4.44 ± 0.47	7.43 ± 0.66	161 ± 43	80 ± 21
NR 2.5 μg/ml	4.39 ± 0.65	7.97 ± 0.75	157 ± 51	474 ± 189
Laser green	4.20 ± 0.66	4.49 ± 0.56	111 ± 36	428 ± 145
	−1% | −6%	7% | −40%	−3% | −31%	492% | 435%
Hybrid DOPC+NR	4.64 ± 1.21/10.1 ± 1.2	9.25 ± 0.62	219 ± 34	112 ± 45
Laser green	5.07 ± 1.14/9.44 ± 1.04	7.43 ± 1.17	122 ± 27	97 ± 47
	9% | −6%	−20%	−44%	−13%

Our FV measurement approach was compared with morphology by tapping mode in liquid using photothermal actuation. While tapping mode morphology in Supplementary Figure SI3A is qualitatively similar to FV morphology (Supplementary Figure SI3B), the quantification shows underestimation of thickness as Δh = 1.4 nm (compare Supplementary Figures SI3D,SI3E). Tapping mode on soft layer is exerting an average non-negligible force, causing a compression not easy to evaluate without force curve approach. We highlight that such compression can be exploited in advanced nanomechanical approaches, such as bimodal AMFM, in order to measure Young’s modulus with high-resolution, as well to correct the morphology adding indentation (Al-Rekabi and Contera, 2018; Liu et al., 2020).

Before introducing Nile red as fluorophore, several control experiments were performed to benchmark selected physical stimulations. Firstly, the influence of continuous measurements was investigated by scanning in FV mode for 3 h on an area (70 μm × 70 μm). Comparing the first and last measurements, no significant differences were detected (see Table 1). In particular, such experiment shows that the formation of holes is reversible and the area of layers is large enough to minimize erosion at the edges. Such reversible character is clearly shown when vertical forces (for example in retracting curve of Figure 1B) decrease below the threshold required to reform the bilayer. Considering that laser irradiation can have a photothermal effect, we measured the mechanical properties of DOPC bilayers gradually increasing temperature of AFM cell up to 41°C. A series of breakthrough maps of phospholipid after temperature increase are shown in Supplementary Figure SI4, showing a slight decrease of F_break_ up to −15%. This is well-documented, as thermal energy decreases the stability of repulsive steric forces in hydrophobic lipid tails. Vertical shrinking and decrease of breakthrough forces was evidenced by Leonenko et al. on phospholipid bilayers including DOPC at 60°C (Leonenko et al., 2004).

Finally, we investigated the effect of laser power focusing the beam on phospholipid bilayer area by maintaining a power density of 35.4 mW/μm^2^ for 30 min. In Supplementary Figure SI5, we compared DOPC control sample with the situation after irradiation, showing only slight decrease of breakthrough forces at −12%. The numerical data are shown in Table 1. In order to avoid systematic uncertainty in model and tip radius determination, we present the final results of all experiments considering control. It is interesting that effect of laser irradiation focused below the probe is similar to environmental cell heating, although no modification of temperature was detected. This behavior is expected to be enhanced when fluorophores, excited by laser light, are employed.

### Revealing the Influence of Fluorophore and Laser Irradiation

Nile red fluorophore, which has the excitation and emission wavelength at 552 and 636 nm respectively, was dispersed in a solution of DMSO/PBS (1:5) prior injection in AFM liquid cell. Considering a volume of 2 ml, we obtained a final concentration of 2.5 μg/ml Nile red and 2.5% solution of DMSO to ensure dispersion in water. Previous studies suggest that the influence of DMSO at a concentration of 2.5% is negligible on phospholipid structure (Hughes et al., 2012; Cheng et al., 2015), moreover we used the standard procedure employed to stain living cells (Rumin et al., 2015).

**FIGURE 4:**
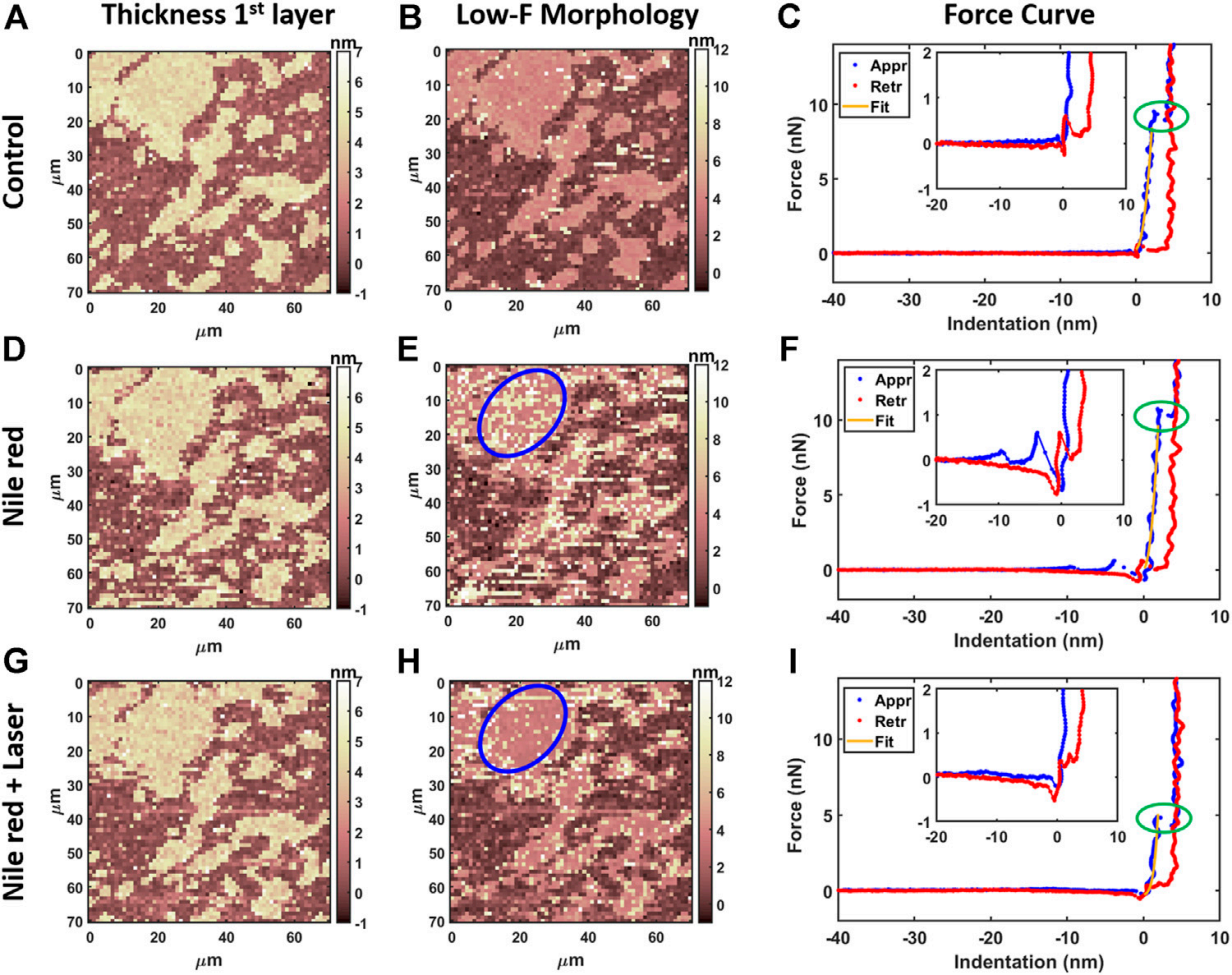
Results of morphological analysis of DOPC partially covering mica using 64x64 force curves. **(A)** Thickness map of first layer and substrate, **(B)** low-force (F < 0.3 nN) morphology and **(C)** examples of approaching (Appr) and retracting (Retr) force curves with fit from Eq. 1 on control DOPC bilayer. Inset represents a zoom near contact point. With the same order of DOPC control **(A–C)**, DOPC after 30 min incubation with 2.5 ug/ml Nile red with DMSO at 2.5% **(D–F)**, and after irradiating probe region with 35.4 mW/μm^2^ laser **(G–I)**. Blue circles in panel e and h are representative of an area where overlayer recedes after laser application. Green ovals in panels **C**, **F**, i highlight breakthrough events.

**FIGURE 5:**
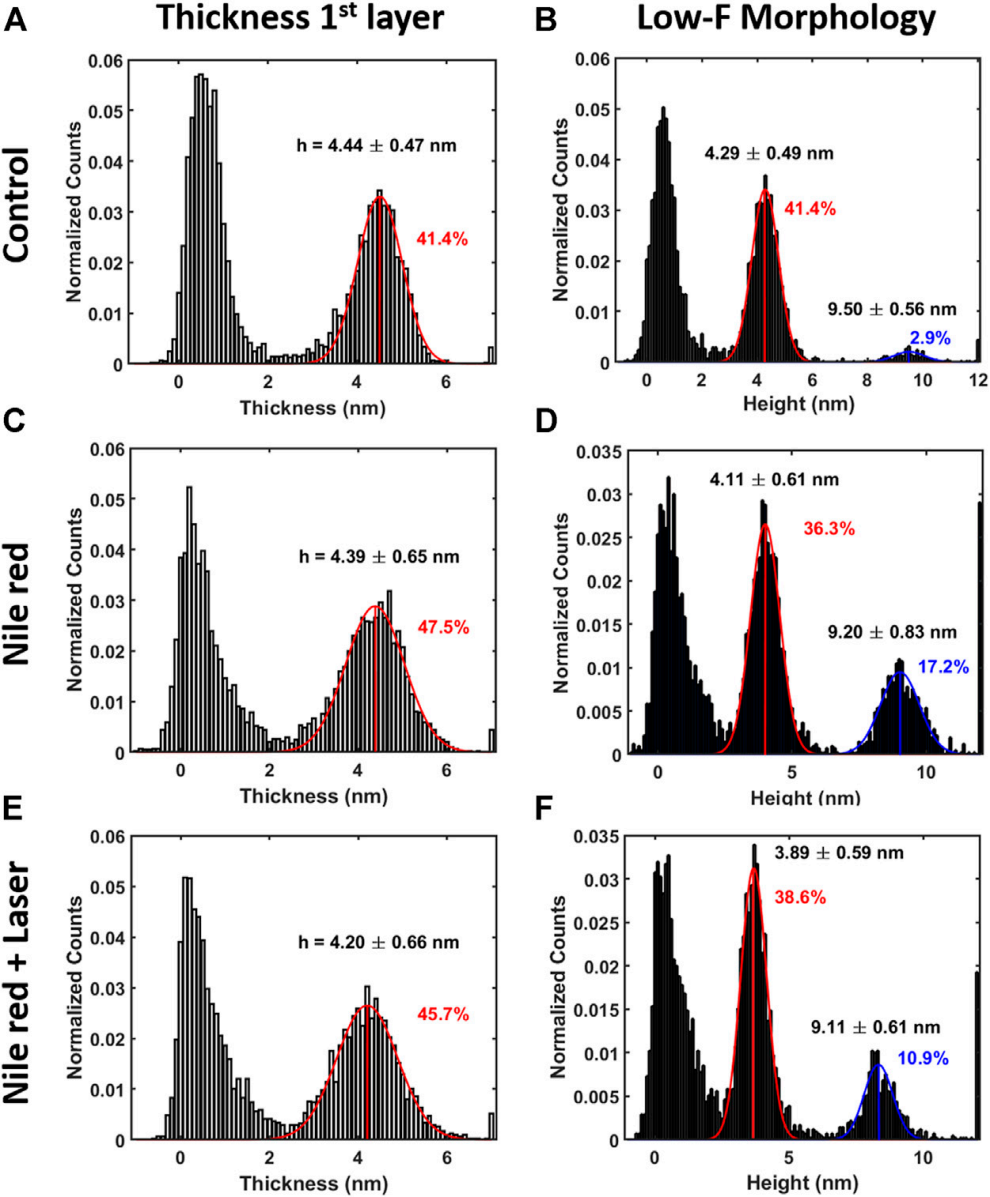
Quantification with histograms and Gaussian fit relative to morphological analysis. **(A)** Thickness histogram of first layer and substrate, **(B)** low-force (F < 0.3 nN) morphology histogram for control DOPC. Same order of histogram of DOPC after 30 min incubation with 2.5ug/ml Nile red with DMSO at 2.5% **(C,D)**, and after irradiating probe region with 35.4 mW/μm^2^ laser **(E,F)**. Gaussian fitting and average are depicted for first (red color) and additional layers (blue color), while percentages are indicative of area coverage (events falling in under the fit curve). Counts of vertical axis are normalized on total number of traces (4096).

AFM measurements were performed after 20 min equilibration upon injection of Nile red. Considering the control DOPC (Figures 4A–C), Figure 4A shows the first layer thickness analysis obtained from force curves (typical example in Figure 4C) using the procedure described in Material and Methods, focusing on bilayer near substrate. Figure 4B is the morphology obtained by detecting an interaction probe-sample with force F = 0.3 nN, showing good overlap with optimized first-layer morphology of Figure 4A. Quantification through histogram is parallelly reported in Figures 5A,B. After injection of Nile red, morphology of first layer is unchanged (Figure 4D) while an increase of thickness is detected in Figure 4E (searching contact point with a low force as F = 0.3 nN) and confirmed quantitatively in Figure 4D. The increase of thickness is relative to oscillations in force curves before contact with first DOPC layer as shown in Figure 4F. With low-force sensitivity, AFM probe is able to detect a weakly bounded overlayer on top of DOPC bilayer. This is suggesting that Nile red molecules are attracted to DOPC bilayer, showing not-negligible interaction and forming weakly bounded aggregates. After injection of Nile red, the force curves on clean substrate are same as control substrate, showing that Nile red molecules are not forming stable interactions with mica surface, as well excluding that surface of probe has contaminations.

After irradiating for 20 min the area under the probe with green laser at 35.4 mW/μm^2^, AFM investigation was performed in the same region, shown in Figures 4G–I. Interestingly, the amount of overlayer detected in Figure 4H decreases noticeably (if compared to Figure 4E), as highlighted in area selected in blue circles. The quantification shown in Figures 5D,F confirms a decrease of coverage of overlayer aggregates from 17.2% to 10.9% peak area. Probably, focusing the laser for several minutes can cause an increase of the mobility of Nile red molecules, leading to displacement of weakly bounded surface aggregates. More dramatic changes are affecting the force curves, evidently decreasing the breakthrough forces (highlighted with green circles in Figure 4) as shown in Figure 4I in comparison with Figures 4C,F.

**FIGURE 6:**
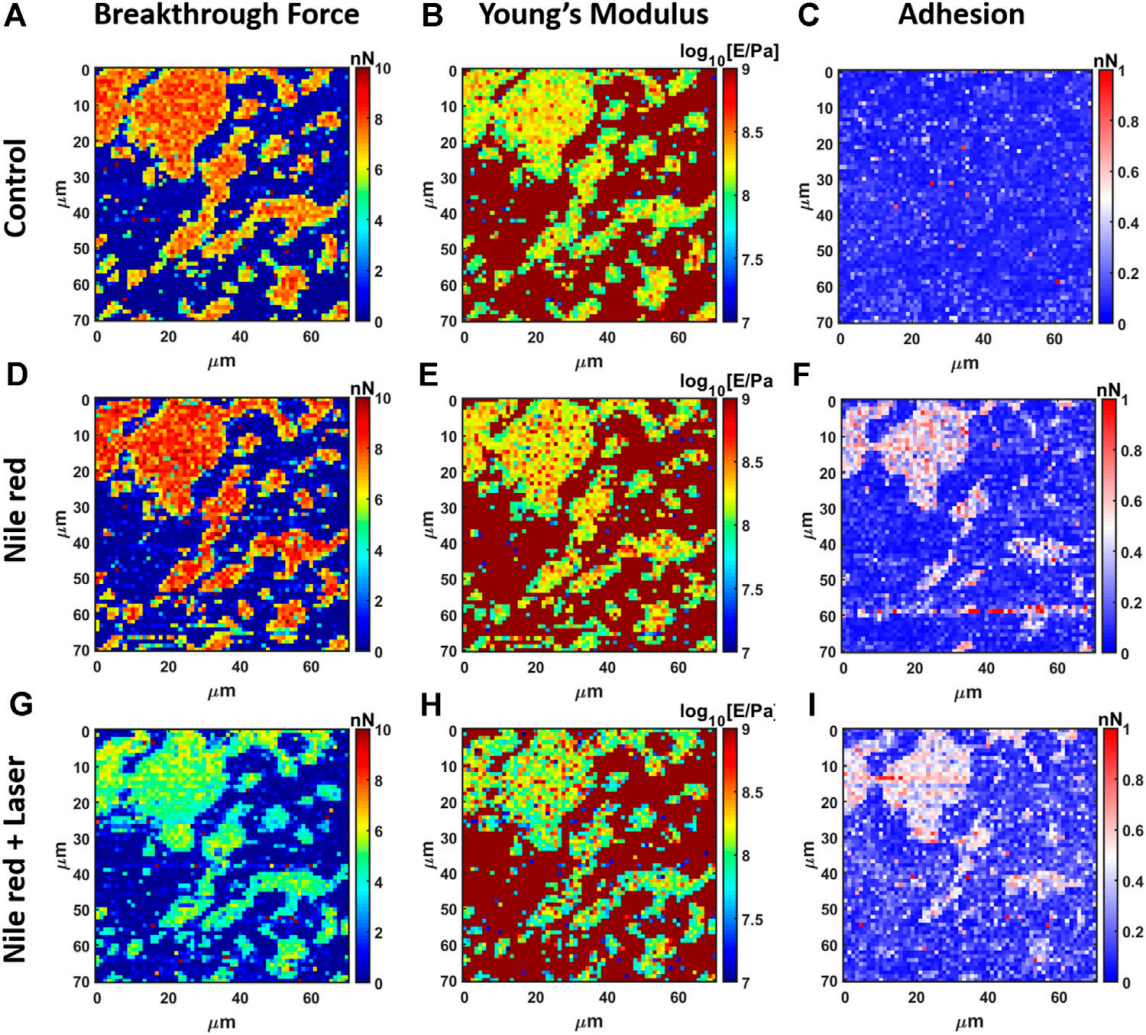
Results of mechanical analysis of DOPC partially covering mica using force curves. **(A)** Breakthrough force map of first layer, **(B)** Young’s Modulus map and **(C)** adhesion map on control DOPC bilayer. With the same order of DOPC control **(A–C)**, DOPC after 30 min incubation with 2.5 ug/ml Nile red with DMSO at 2.5% **(D–F)**), and after irradiating probe region with 35.4 mW/μm^2^ green laser **(G–I)**.

**FIGURE 7:**
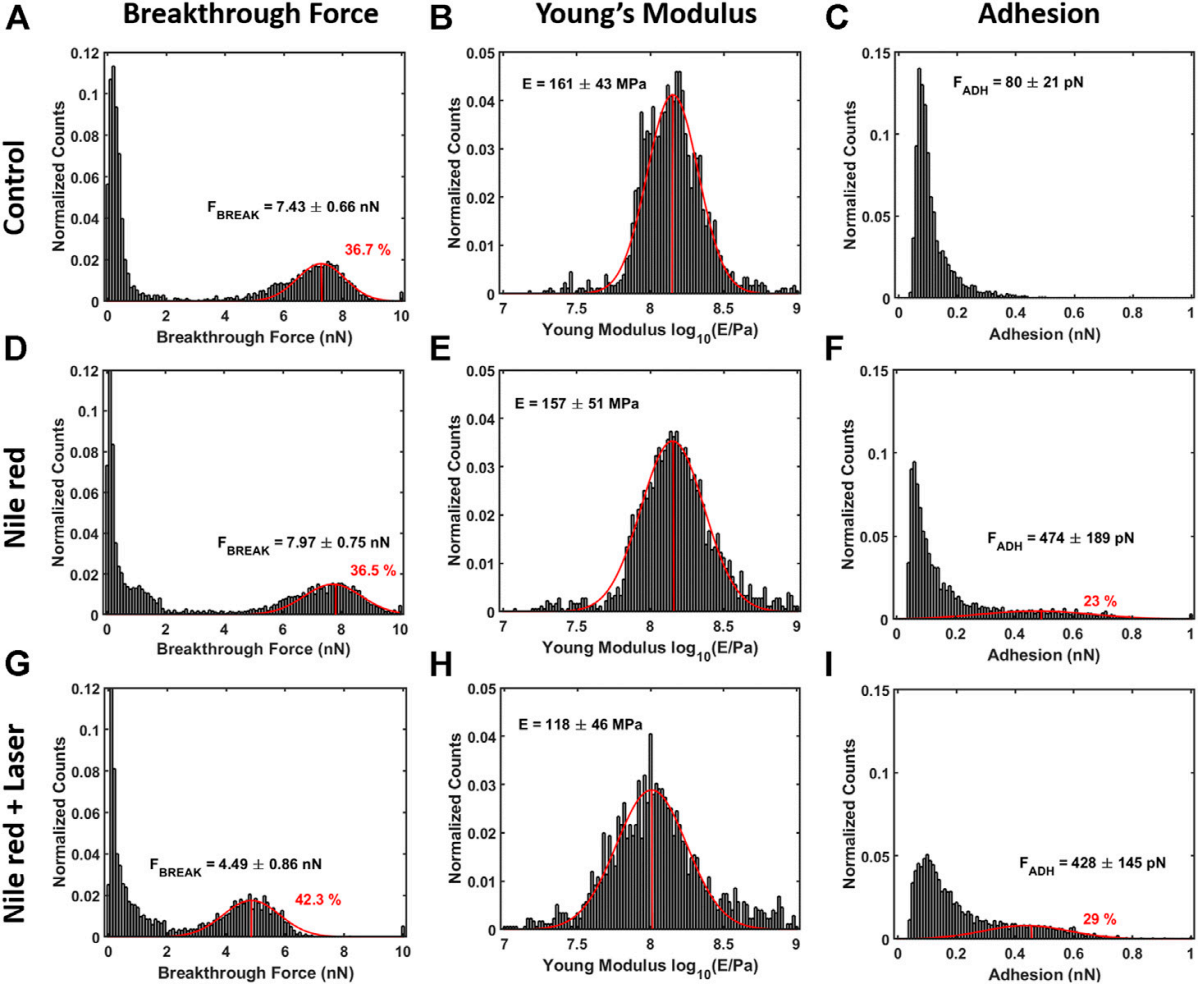
Quantification with histograms and Gaussian fit relative to mechanical analysis. **(A)** Breakthrough force histogram of first layer, **(B)** Young’s Modulus histogram and **(C)** adhesion histogram on control DOPC bilayer. With the same order of DOPC control **(A–C)**, DOPC after 30 min incubation with 2.5ug/ml Nile red with DMSO at 2.5% **(D–F)**), and after irradiating probe region with 35.4 mW/μm^2^ green laser **(G–I)**. Gaussian fitting and average are depicted for first and additional layers, while percentages are indicative of area coverage (events falling in under the fit curve). The peaks around zero breakthrough force in **(A,D,G)** reflect the fact that some parts of the mica surface were not covered by DOPC bilayer. Counts of vertical axis are normalized on total number of traces (4096).

The nanomechanical analysis is evidencing modifications related to stability and elasticity. Considering the control DOPC, we show the maps for breakthrough force required to break the DOPC bilayer in Figure 6A, the Young’s Modulus in Figure 6B and the adhesion force between probe and layer in Figure 6C. Figure 7 shows the quantification through histogram relative to Figure 6, while data are presented and compared in Table 1. For instance, after adding Nile red to the solution, the adhesion increases noticeably in agreement with the presence of a weakly bound overlayer that can trap the AFM probe after closing the layer during the retract phase (see adhesion map Figures 6F,I compared with control Figure 6C). Also in agreement with idea of weakly bounded overlayer of Nile red, the breakthrough force threshold slightly increases, in fact, similar to multilayer structures, more force is required to displace laterally the overlayer before final breakthrough event (Relat-Goberna et al., 2017).

After 30 min laser irradiation, the effect on layer mechanics is very noticeable, exhibiting a decrease of force required to breakthrough (−40%, Figures 6G, 7A,G decrease of Young’s modulus (−31%, Figures 6H, 7H). The synergic effect of fluorophore and laser may be an indication of laser energy absorption by Nile red, subsequent relaxation (part of incident energy dissipated non-radiatively) and final decrease of ordering/stability reflected by mechanics of DOPC phospholipid layer.

The experiments of DOPC bilayer interacting with Nile red and green laser were repeated, maintaining the same conditions but changing AFM probe. In this case, the value of radius may have large errors leading to different absolute values in breakthrough force and Young’s modulus.

Particularly, the experiment was performed 3 times showing the same behavior. While morphological analysis shows negligible modifications, breakthrough force has slight increase after adding Nile red and noticeable decrease after laser application. An overlayer is visualized from force curves after staining, while disappearing after laser application. The two additional independent experiments using different probes are in supporting information respectively in Supplementary Figures SI6, SI7, while data are collected in Supplementary Table SI1.

### Increasing Concentration of Nile Red

To further explore the combined effect of amphiphilic fluorophore Nile red and laser on DOPC bilayer, we produced a hybrid structure by mixing 10 mg/ml DOPC with 2.5 μg/ml Nile red in chloroform before dispersing in water. While in previous experiments we experience interaction of Nile red at the most exterior phospholipid leaflet, the objective is to produce a hybrid system including Nile red homogeneously in both leaflets. After chloroform evaporation and rehydration in PBS, hybrid liposomes in solution are red indicating Nile red is incorporated in apolar lipidic environment. Nile red is blue/purple in a polar environment such as water and quickly aggregating. Compared with previous experiments where same concentration of Nile red is dispersed in PBS, we expect the effective quantity of Nile red within the hybrid system is higher.

After deposition on mica, AFM morphology shows DOPC + Nile red hybrids formed irregular, fragmented layers (oppositely to pure DOPC) and double bilayers were often encountered, as shown in Supplementary Figure SI8. In average, the breakthrough force is higher than pure DOPC single layer, as expected from multilayer systems showing the layer near the substrate exhibiting higher stability (see Supplementary Figure SI8B for map and Supplementary Figure SI8C examples of force curves on single or double bilayer). After several measurements, we have evidenced that double bilayer of hybrid structure is the most stable. It may be possible that Nile Red molecules added before micellization in water can favor the formation and stabilization of multilayer structures.

Laser is causing large delamination of layers and reorganization of material. The hybrid structure is becoming very movable, while the probe is probably influencing the displacement of layers by lateral drags. Supplementary Figures SI9D,SI9E show the layers broken and certain amorphous accumulation near the scan area border (evidenced by red circles). By comparing the quantification through histograms in Supplementary Figure SI9, the second bilayer almost disappears and the first greatly reduces in dimensions. The breakthrough force and Young’s modulus decrease about −20% and −44%, respectively in comparison with control. When mixed before micellization in water, we speculate a stronger interaction between hydrophobic Nile red and core of lipid tails.

### DOPC Interacting with Other Dyes

The investigation of model membrane interacting with different dyes and lasers is important to understand how much the synergy between dye excitation and mechanical modifications can be generalized.

For example, we performed AFM nanomechanical experiments on DOPC membrane interacting with different dyes and lasers. If not stated otherwise we always use the experimental conditions described in Material and Methods.

First, we used CytoTracker deep red, a staining agent, to visualize cytosol for the purpose of tracking cells’ movements. This dye is more hydrophilic than Nile Red; not specific for cell membrane, but membrane permeable. Therefore, in a model system such as supported bilayer with staining solution always in contact, a certain interaction was expected. Using operative concentration of 2.5 μg/ml no modification was evidenced. Only increasing concentration 10 times (25 μg/ml) a decrease of breakthrough force was showed similarly to Nile red. The corresponding results are shown in Supplementary Table S2; Supplementary Figure SI10.

Secondly, we used HOECHST, a common staining agent for DNA molecules in order to visualize nucleus in confocal measurements. The dye is amphiphilic, not specific for cell membrane but permeable. Here, using operative staining concentration of 2.5 μg/ml we noticed a large deposit forming on top of DOPC layer causing difficulties in AFM measurement. The thickness doubled the breakthrough force, while the adhesion force increased 100 times, evidencing a complex of DOPC/dye molecules strongly attracting AFM probe. The laser application (same power as in main text but blue color) was dramatic, removing all material from surface within 1 min. The corresponding results are shown in Supplementary Table S2; Supplementary Figure SI1, in particular we show adhesion signal as the most significant.

Hydrophilic molecules require higher concentration to modify the membrane, while we speculate that they can have more influence on their target (proteins in cytosol). Amphiphilic molecules such as HOECHST can have more effect on membrane (especially after long time of continuous interaction). For this reason, standard protocols for HOECHST staining are requiring to remove excess of dye by washing after short time (15 min).

Finally, we highlight that the effects we observed here are dependent on the nature of the system. While a general photooxidation is expected when using focused laser, the intensity of mechanical modifications depends on the interaction between dye molecules and biological target, therefore additional investigations using confocal AFM mechanics could help in determining which system should be treated with more care.

## Discussion

Fluorescence microscopy, especially the laser confocal scan microscopy, raises the concern of biologically relevant modifications induced by irradiated laser, particularly in the presence of dye molecules. Whether these effects can affect the mechanical properties becomes critical for membrane integrity during live measurements. Hidden events in standard fluorescence microscopy can be detected by implementing optomechanical correlative microscopy analysis. Firstly, by controlling the applied force during analysis, we detected Nile red fluorophores forming weakly bounded composite structures on top of phospholipid layer near substrate. Weakly bounded layer is visible in force oscillation in Figure 4F as well increase of adhesion force during retracting. These oscillations are also detected by AFM force spectroscopy when weakly bounded molecular electrolytes are attracted to solid surface electrode (Sakai et al., 2015; Cui et al., 2016; Griffin et al., 2016). In this framework, phospholipid mixtures were investigated using rhodamine-based phospholipid as fluorescent probe to visualized raft structures (Ayuyan and Cohen, 2006). Excitation of fluorophore induced photooxidation of DOPC in a three component lipid mixture, evidenced by the formation of large raft-like structures. While a substantial amount of DOPC may be converted in lipid peroxides easily visible by fluorescence microscopy, mechanical properties were never investigated. Nanomechanics by AFM was used in this work to unveil modification not easily detected during fluorescence microscopy. In fact, we revealed that the degradation of mechanical properties of model phospholipid membranes is driven by synergic presence of bounded fluorophores and focused laser irradiation. Similar to modification induced by temperature rise (Leonenko et al., 2004; Garcia-Manyes et al., 2005), we expect laser energy transfer between fluorophores and biomembranes is at the base of this modification. Instead, it was evidenced that fluorescence intensity is decreasing dependent with temperature increase for DOPC and Nile red system (Halder et al., 2018). Such effect was explained as temperature related increase of flexibility and consequent water penetration, leading to partial Nile red quenching. Beside quenching, we expect the modification of mechanical properties induced by combined action of laser and fluorophore will lead to additional deterioration of fluorescence performance. Conversely, the possibility to tailor fluorescence and photothermal relaxations for near-infrared fluorophores was recently explored by Gao et al. (Gao et al., 2019) In particular, fluorescence imaging can enhance intraoperative guidance, at the same time allowing photothermal ablation therapy for cancer lesions. In confocal experiments, the signal/noise ratio can be improved by increasing the quantity of staining agent or laser light intensity. While this strategy is proven effective, photo quenching is often encountered, moreover, we now highlight how the structural equilibrium of phospholipids can break upon irradiation if relative quantity of fluorophore is high. As shown in the hybrid system, photothermal energy conversion may be responsible for degradation of mechanical properties and increased mobility stressed by relatively higher quantity of accumulated Nile red. Similar behavior (increased mobility and fluidity, displacement of layers during measurements) are observed when using cationic surfactants strongly interacting with lipid tails (Lima et al., 2013; Redondo-Morata et al., 2014).

## Conclusion

This work highlights the analytical power provided by AFM to obtain subtle yet important information, which are otherwise hard to detect, in correlative microscopy as well as the necessity in taking photothermal effects into account when using fluorophores in examining the behavior of cell membranes. Indeed, a synergistic effect of focused laser and Nile red fluorophore interacting with DOPC model membrane was detected by AFM nanomechanics, showing remarkable decrease of stability and elasticity of supported bilayers. This effect is comparable to that caused by elevated temperature, suggesting the critical role of local heat transfer (within the membrane) induced by excitation/relaxation of Nile red fluorophores in this process. The equilibrium between radiative (fluorescence) and non-radiative (lattice vibrations) relaxations is worth investigating in order to evaluate the extent of prospective applications when using multi-modal nanoparticles able to switch fluorophore and photothermal characteristics. Our results are accompanied by a robust and reliable methodology to implement correlative AFM mechanics and optical microscopy, as well a routine of custom data analysis. While we investigate phospholipid bilayers influenced by excited Nile red, the scope of application of this methodology can be extended to other biosystems where synergic action of fluorophore and laser may play important role in nanomechanical modifications.

## Data Availability

The raw data supporting the conclusions of this article will be made available by the authors, without undue reservation.
