# Supplementary material for: Unveiling a Hidden Event in Fluorescence Correlative Microscopy by AFM Nanomechanical Analysis
Source: Front Mol Biosci. 2021 May 6;8:669361. doi: 10.3389/fmolb.2021.669361 (PMC8136518; doi:10.3389/fmolb.2021.669361)
Supplement: Supplementary file 2 [file DataSheet1.docx]

**Unveiling a hidden event in fluorescence correlative microscopy by AFM nanomechanical analysis**

Supplementary Material

# Focusing the laser

Figure S1 shows the calculation of optical power density when the laser spot is focused on the probe region using objective lens. In our case, PBS is used, which has the refractive index similar to water. 40X objective lens with NA of 0.6 is used and we assume that the full NA of the objective lens is utilized in our case. When the laser power was set to be 200 mW, the final estimated value of output optical power density is around 700 mW/μm^2^. Due to the NA utilized in actual is less than 0.6, the actual output optical power density is less than 700 mW/μm^2^.


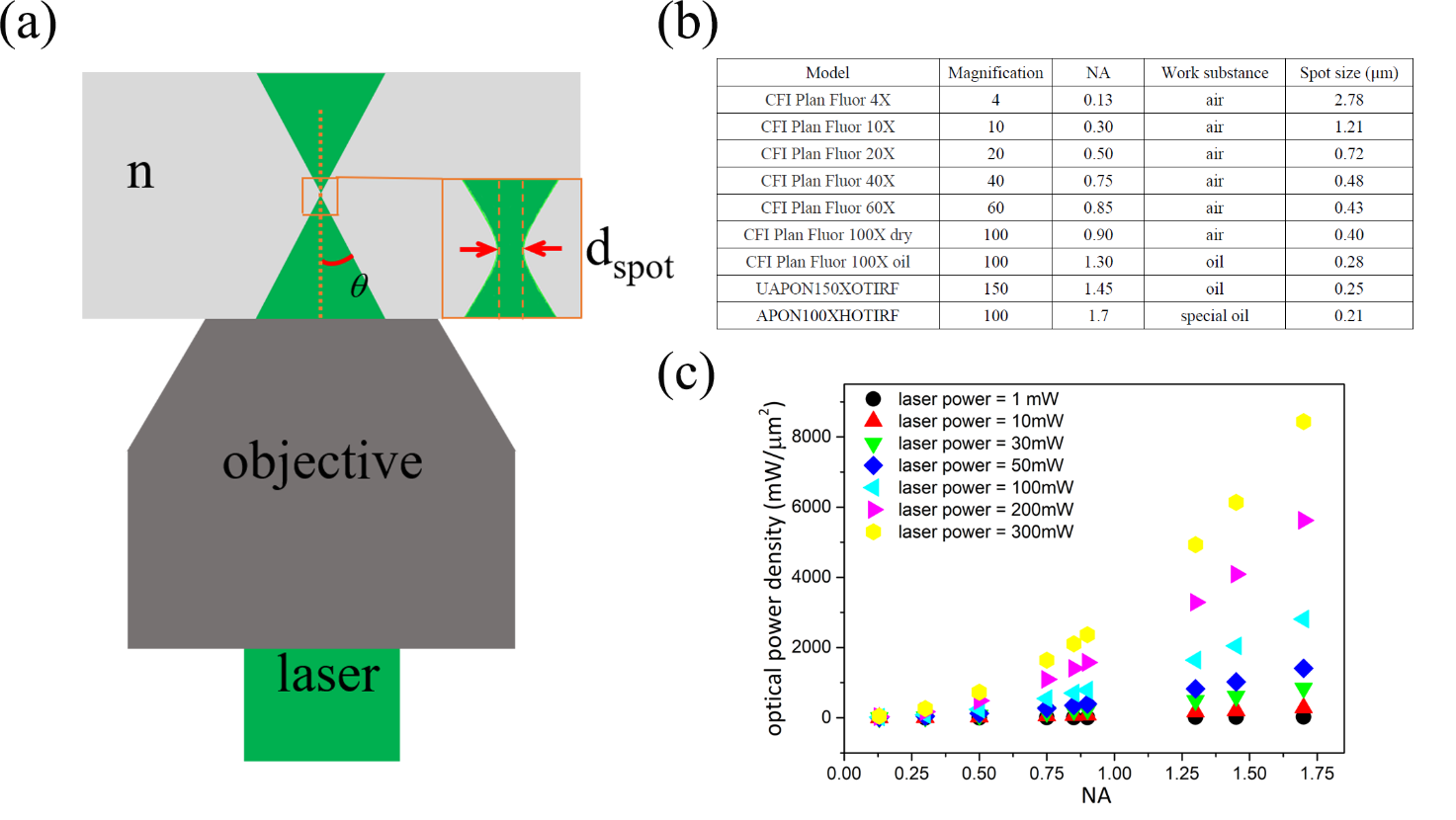


Figure SI1. Calculation of the output power density. (a) The process of focusing a laser beam, spot size can be calculated according to equation (2). (b) Calculation of the spot sizes for objective lenses in the table: part of CFI Plan Fluor series objective lenses (Nikon, these objectives lenses are super in fluorescence live-cell imaging with high quality phase difference images) and two special objective lenses (Olympus) respectively owning high NA and high magnification. (c) Calculation of optical power density for objective lenses in (b) according to equation (3).

The resolution of confocal microscopy is defined as full width at half maximum (FWHM) of point spread function. According to Olympus officail website^[1]^, the empirial formula about FWHM is expressed as:

$FWHM=\frac{0.4\lambda}{NA}$ (1)

Combining equation (1) and the relationship between FWHM and the spot size, we can get:

$d_{spot}=\frac{2*FWHM}{\sqrt{2ln(2)}}\approx\frac{0.68\lambda}{NA}$ (2)

where $\lambda$ (the wavelength of laser we use) is 532 nm; NA (numerical aperture, obtained from datasheet of objectives) can be written as $nsin(\theta)$ and n is the refractive index of work substance; we assume that the transmittance is 100% (actually around 85-95%). Optical power density can be written as

$W_{density}=\frac{4W_{laser}}{\pi d_{spot}^{2}}$ (3)

where $W_{laser}$ is the output power of laser and $d_{spot}$ is the spot size.

# Supplementary Figures and Tables


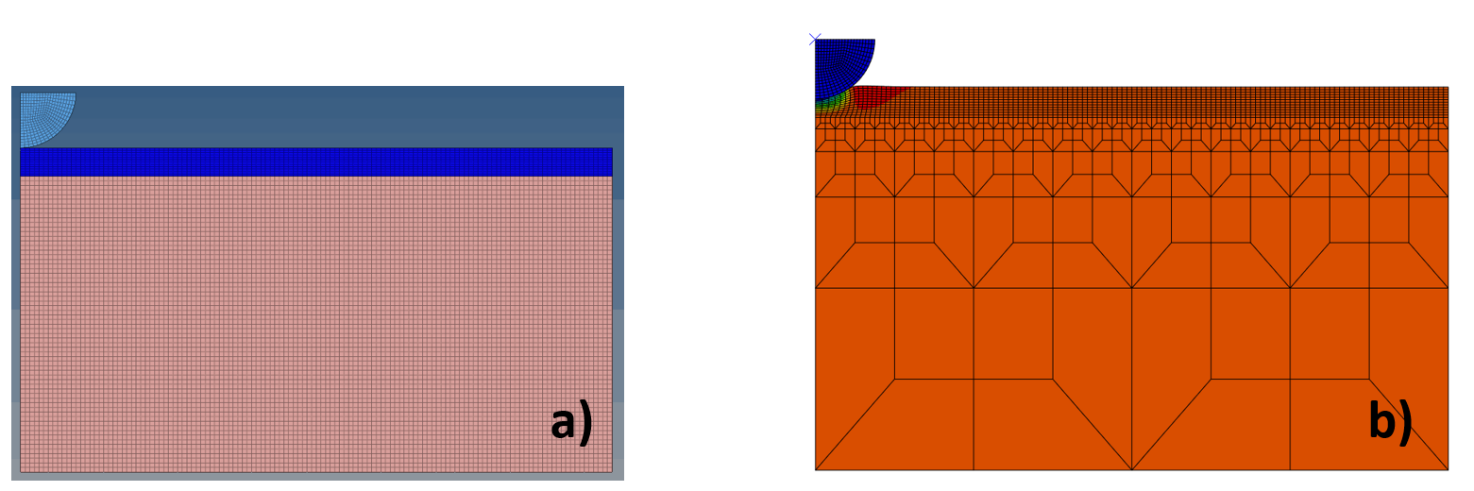


Figure SI2. Construction of FEM of 10 nm radius probe indenting 5nm layer supported on Silicon. a) the element and materials modelling b) stress field along vertical indentation direction.


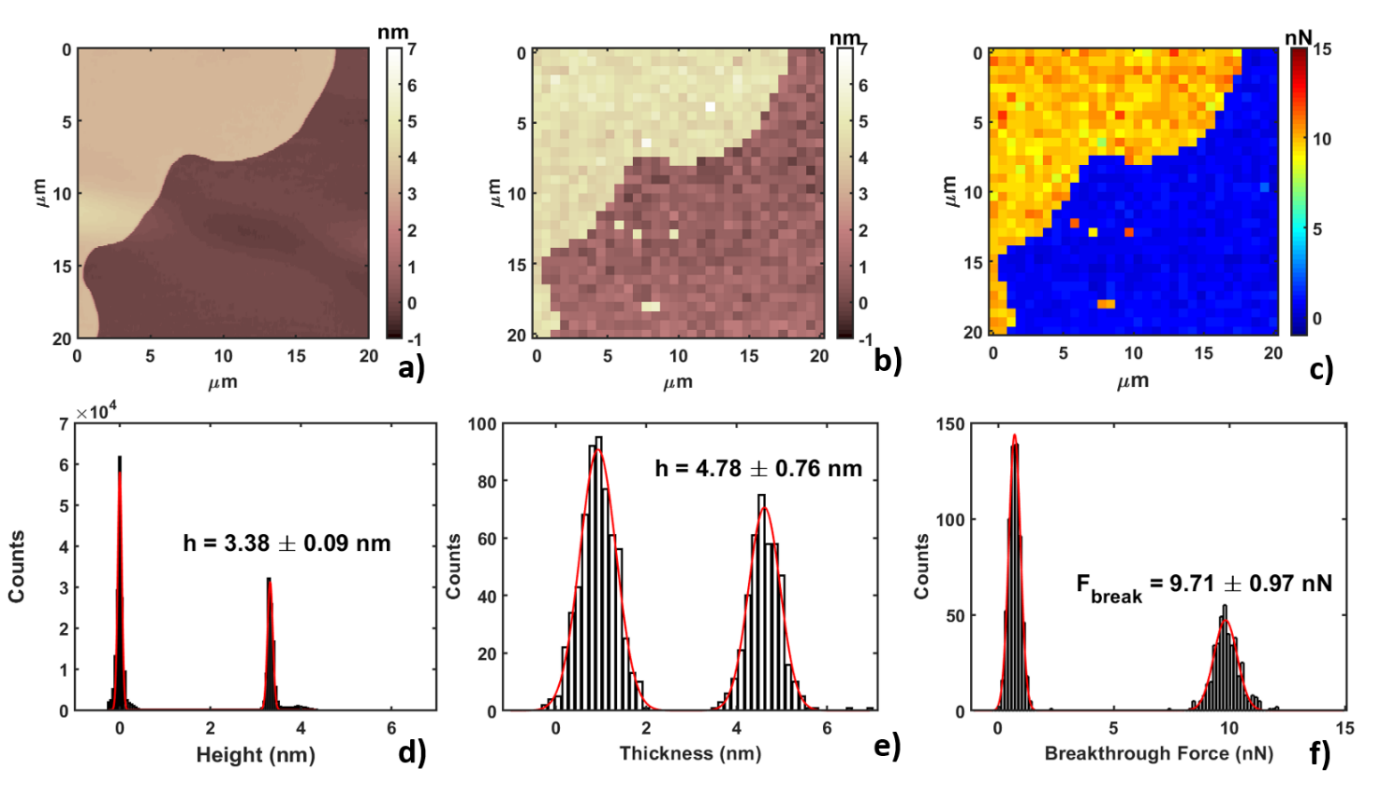


Figure SI3. Tapping mode and Force Volume in comparison. Example of a) standard tapping mode morphology in liquid using blue drive photothermal actuation, b) thickness map from Force Volume in same region, c) breakthrough force map. The respective histograms relative to maps a-c are shown below in d-f, showing Gaussian fitting and final results.


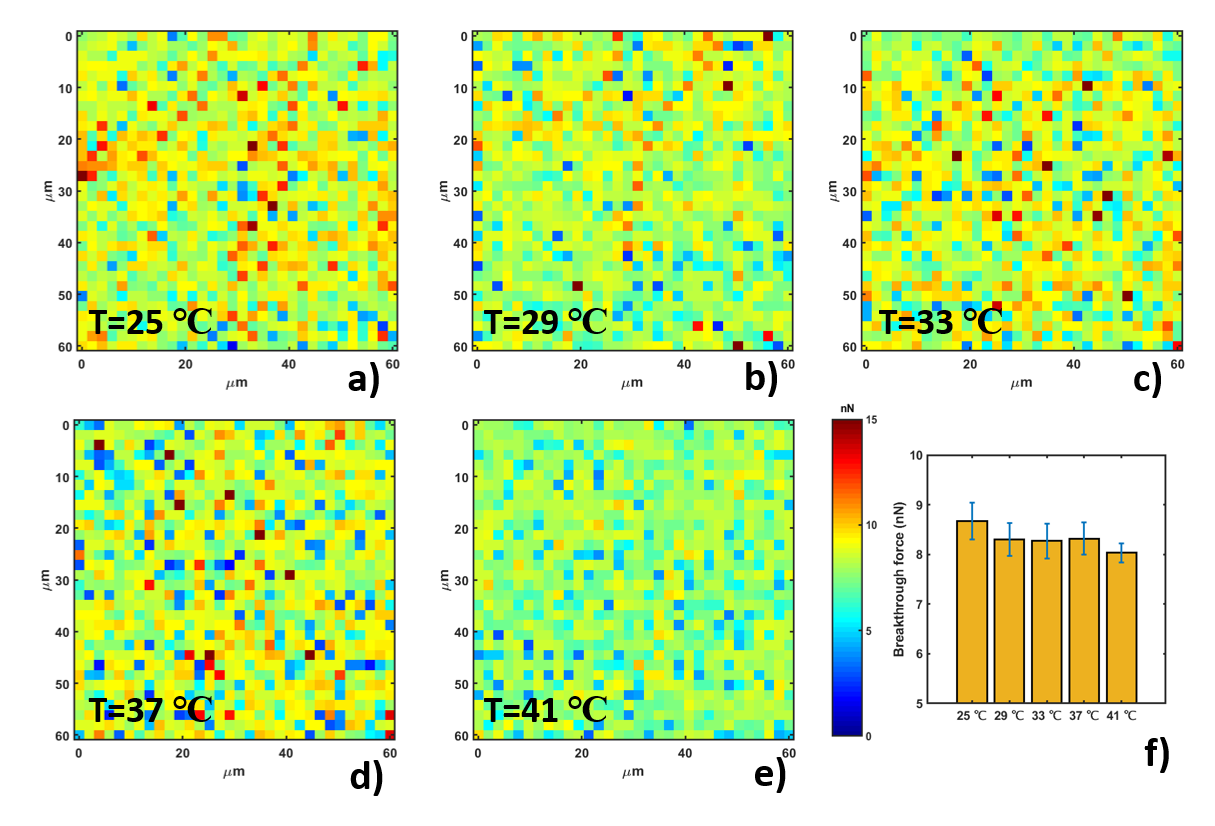


Figure SI4. The effect of global temperature. Breakthrough force map for a) 25 ℃, b) 29 ℃, c) 33 ℃, d) 37 ℃, e) 41 ℃. The average and error values from histograms are collected in f).


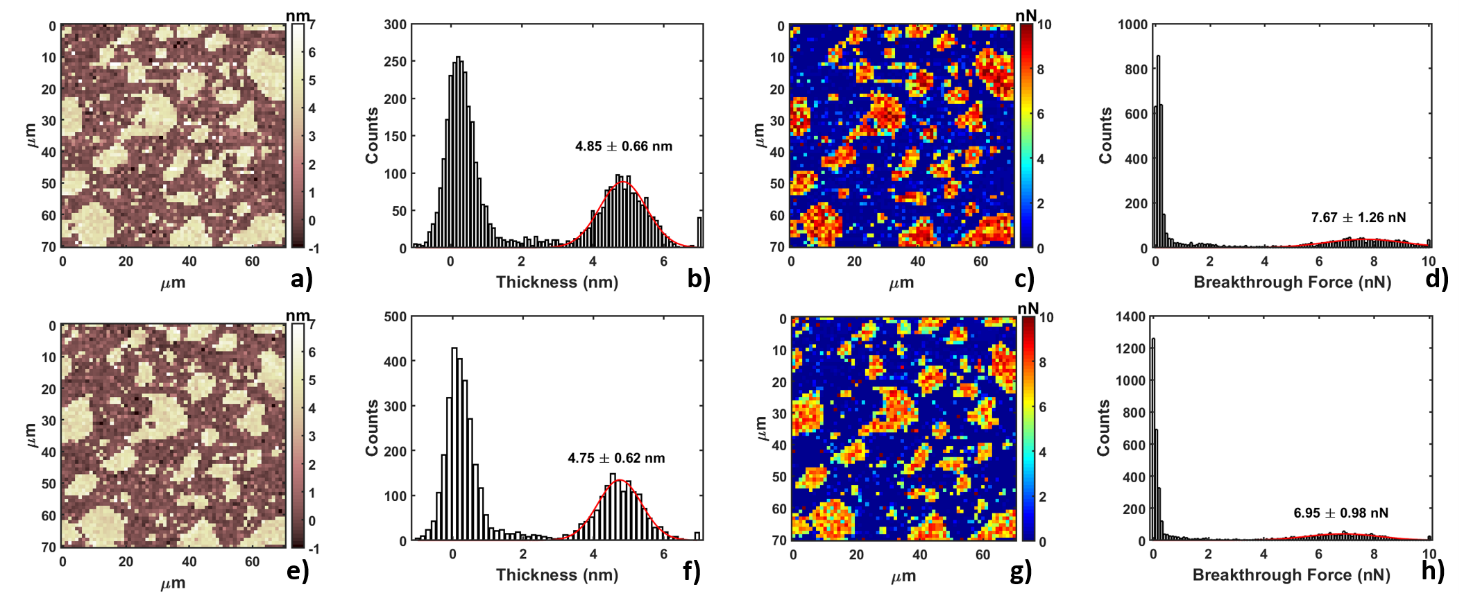


Figure SI5. Laser irradiation. a) Morphology map, b) morphology histogram, c) breakthrough force map d) breakthrough force histogram for DOPC supported on mica (control). Using the same order of graphs a-d, we present the morpho-mechanical analysis after 30 min laser irradiation, showing only a slight (not significant) decrease of breakthrough force is detected. All the other morpho-mechanical parameters are unaltered. (a-d) control, (e-h) with laser focused on probe.

| **condition** | **Thickness (nm)** | **Breakthrough (nN)** | **Young's Modulus  (Mpa)** | **Adhesion (pN)** |
| --- | --- | --- | --- | --- |
| Probe #2 | | | | |
| control | 5.51 ± 0.65 | 8.60 ± 1.38 | 105 ± 14 | 67 ± 9 |
| NileRed 2.5μg/ml | 5.09 ± 0.51 | 9.25 ± 0.65 | 108 ± 9.5 | 169 ± 49 |
| Laser green | 4.93 ± 0.47 | 6.15 ± 0.41 | 73 ± 10 | 112 ± 23 |
|  |  |  |  |  |
| Probe #3 | | | | |
| control | 4.70 ± 0.54 | 6.34 ± 0.70 | 98 ± 7 | 64 ± 7 |
| NileRed 2.5μg/ml | 4.96 ± 0.59 | 8.14 ± 0.53 | 104 ± 17 | 145 ± 6 |
| Laser green | 4.89 ± 0.53 | 4.66 ± 1.31 | 68 ± 8 | 53 ± 2 |

Table S1 Summary of additional experimental results of DOPC supported bilayers interacting with Nile red and laser radiation.


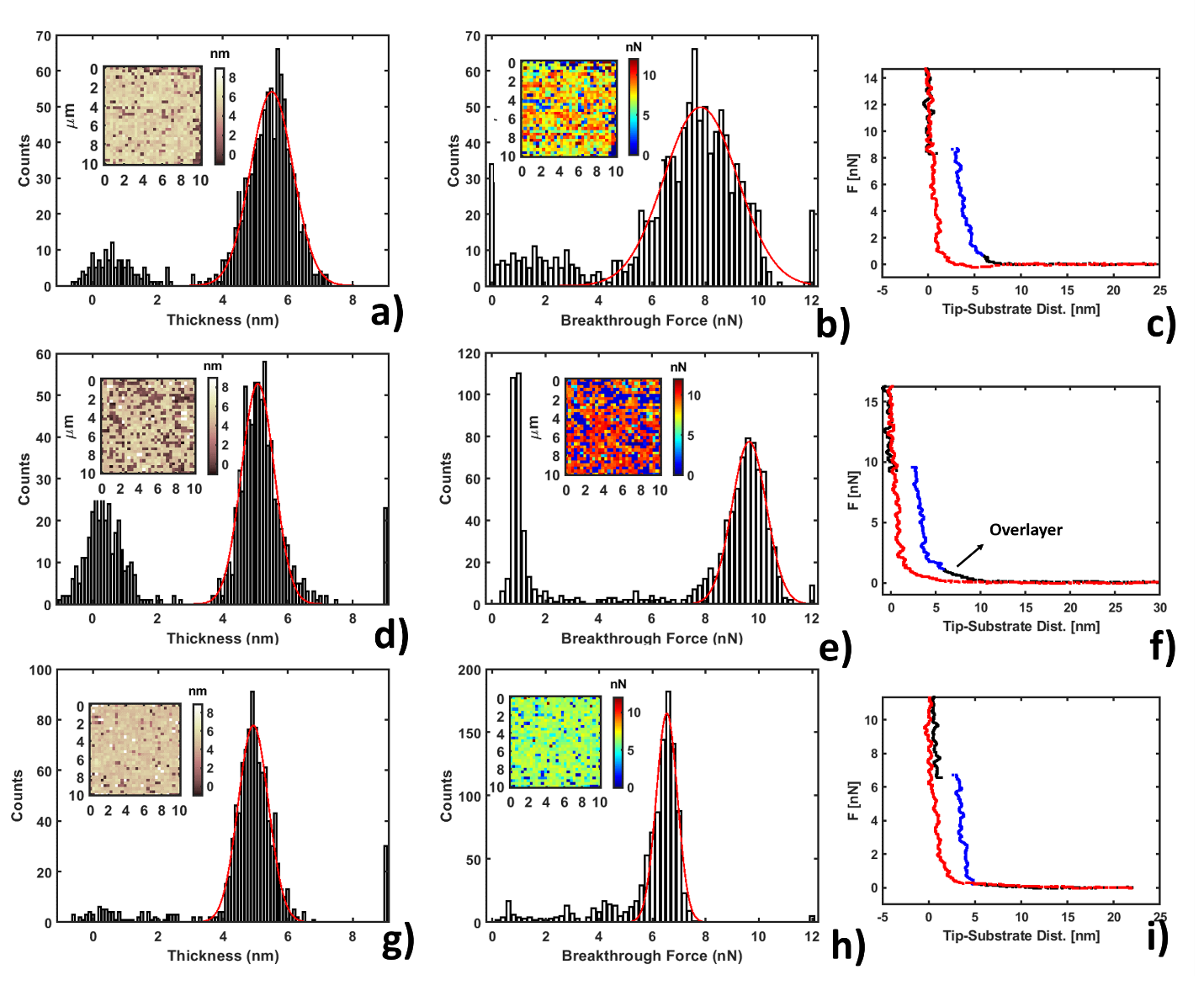


Figure SI6. Additional measurements of DOPC interacting with Nile red. a) Thickness histogram of DOPC bilayer with correspondent topography image. b) Breakthrough force histogram with correspondent map; c) Example of force curve on bilayer with approaching (blue) and retracting (red). With the same order of DOPC control (a-c), DOPC after 30 min incubation with 2.5 ug/ml Nile red with DMSO at 2.5% (d-f), and after irradiating probe region with 35.4 mW/μm^2^ green laser (g-i).


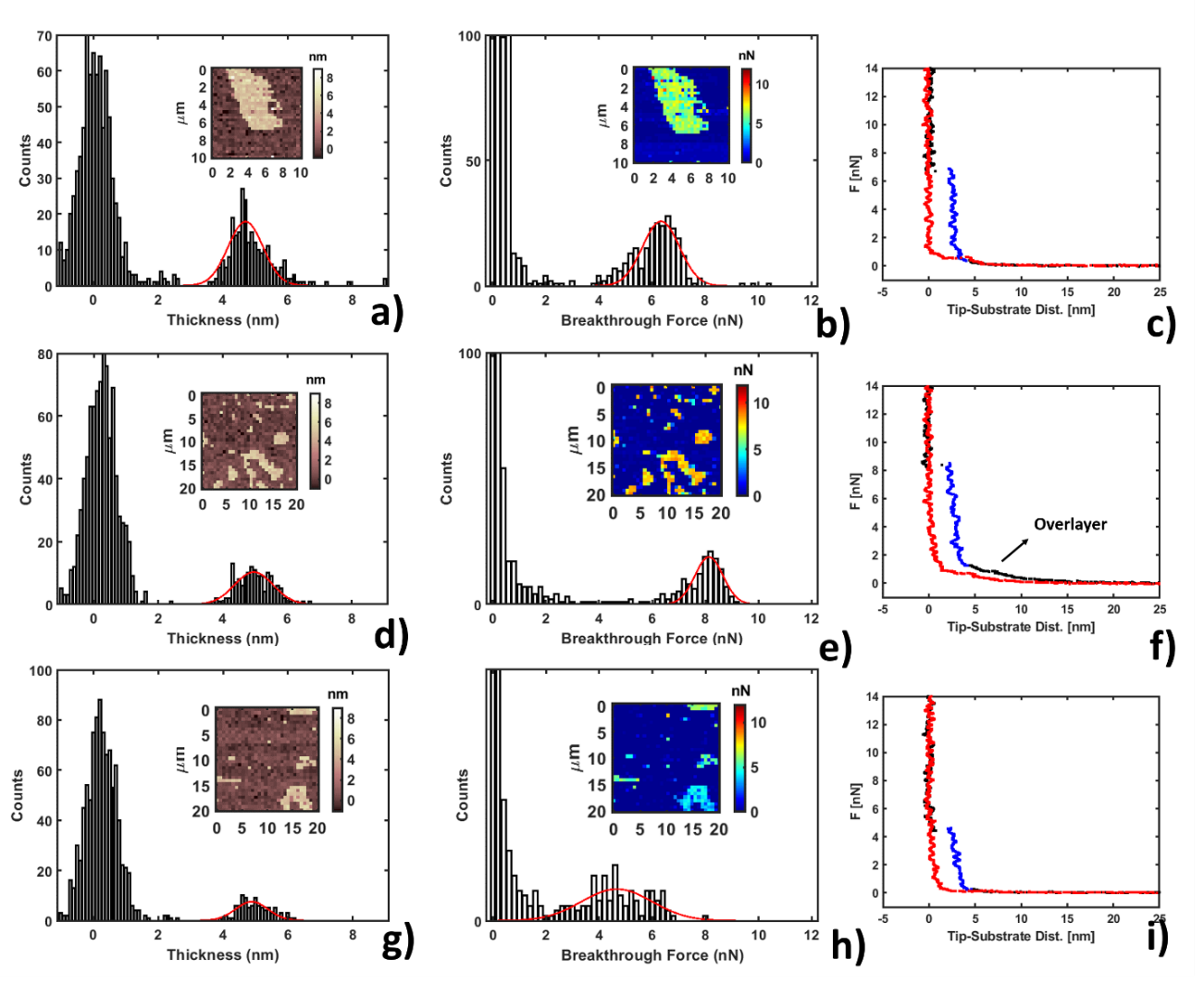


Figure SI7. Additional measurements of DOPC interacting with Nile red. a) Thickness histogram of DOPC bilayer with correspondent topography image. b) Breakthrough force histogram with correspondent map; c) Example of force curve on bilayer with approaching (blue) and retracting (red). With the same order of DOPC control (a-c), DOPC after 30 min incubation with 2.5 ug/ml Nile red with DMSO at 2.5% (d-f), and after irradiating probe region with 35.4 mW/μm^2^ green laser (g-i).


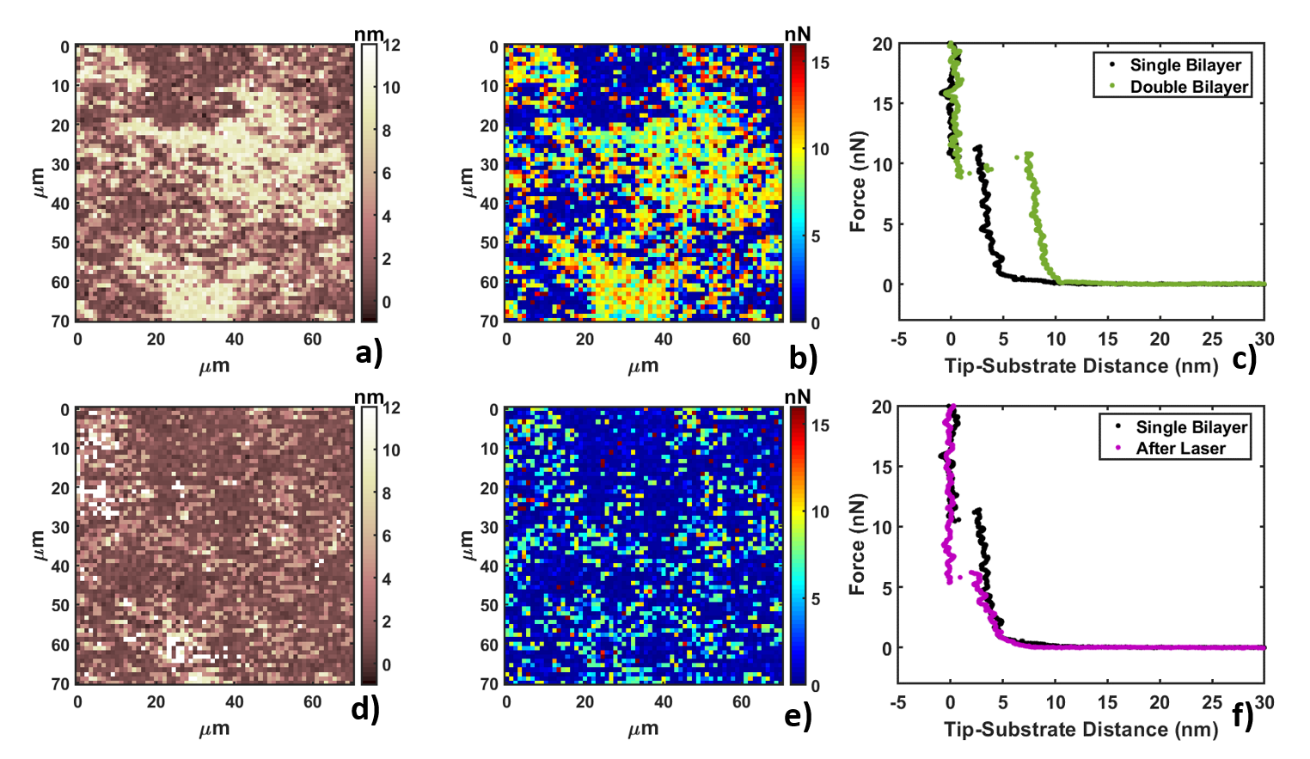


Figure SI8. Hybrid structure of DOPC+ Nile red. a) morphology, b) breakthrough force map and c) examples of force curves of hybrid before application of laser irradiation. Using the same order, (d-f) hybrid structure after 20 minutes application of 35.4 mW/mm^2^ green laser irradiation.


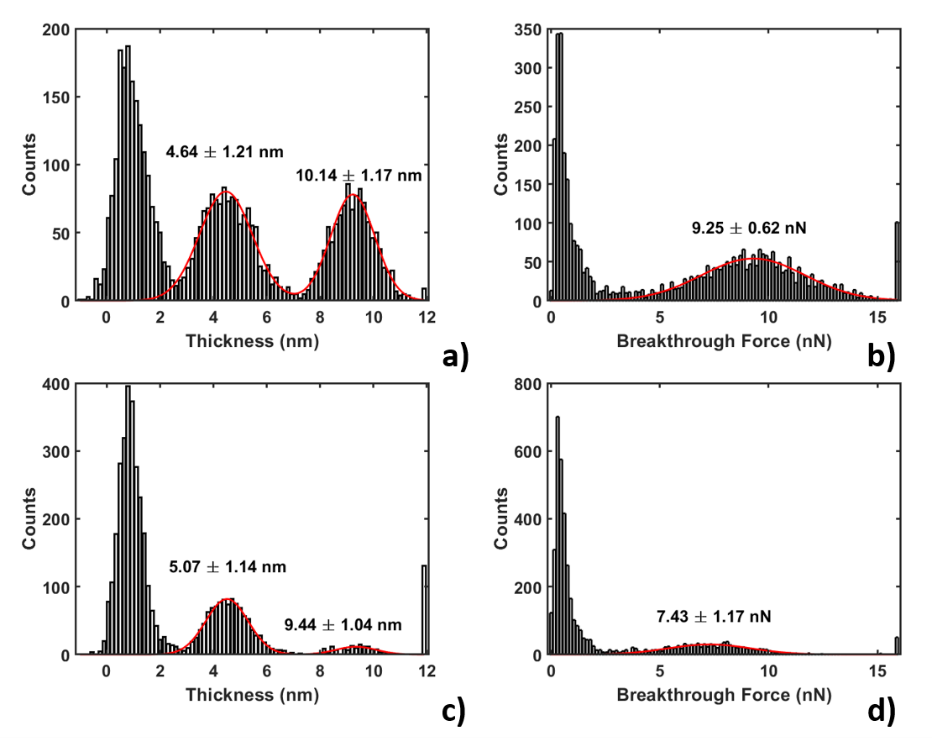


Figure SI9. Quantification with histograms and Gaussian fit relative to figure 5. a) morphology histogram, b) breakthrough force histogram of hybrid before application of laser irradiation. Using the same order, (c - d) hybrid structure after 20 minutes application of 35.4 mW/μm^2^ green laser irradiation.

ADDITIONAL DATA

| **condition** | **Thickness (nm)** | **Breakthrough (nN)** | **Young's Modulus  (Mpa)** | **Adhesion (pN)** |
| --- | --- | --- | --- | --- |
| CytoTracker deep red 25 mg /ml | | | | |
| control | 4.95 ± 0.67 | 6.32 ± 0.69 | 66 ± 8 | 62 ± 7 |
| CytoTracker | 4.99 ± 0.62 | 6.55 ± 0.27 | 63 ± 7 | 66 ± 6 |
| Laser green | 4.93 ± 0.47 | 4.72 ± 1.89 | 55 ± 6 | 65 ± 7 |
|  |  |  |  |  |
| HOECHST 2.5 mg /ml | | | | |
| control | 4.70 ± 0.54 | 6.34 ± 0.70 | 146 ± 31 | 54 ± 7 |
| HOECHST | 11.26 ± 0.89 | 14.14 ± 1.52 | 56± 27 | 11754 ± 1375 |
| Laser blue | no layer | no layer | no layer | 101 ± 121 |

Table S2 Summary of additional experimental results of DOPC supported bilayers interacting with CytoTracker deep red 25 mg/ml, HOECHST 2.5 mg/ml and respective laser radiations (green and blue).


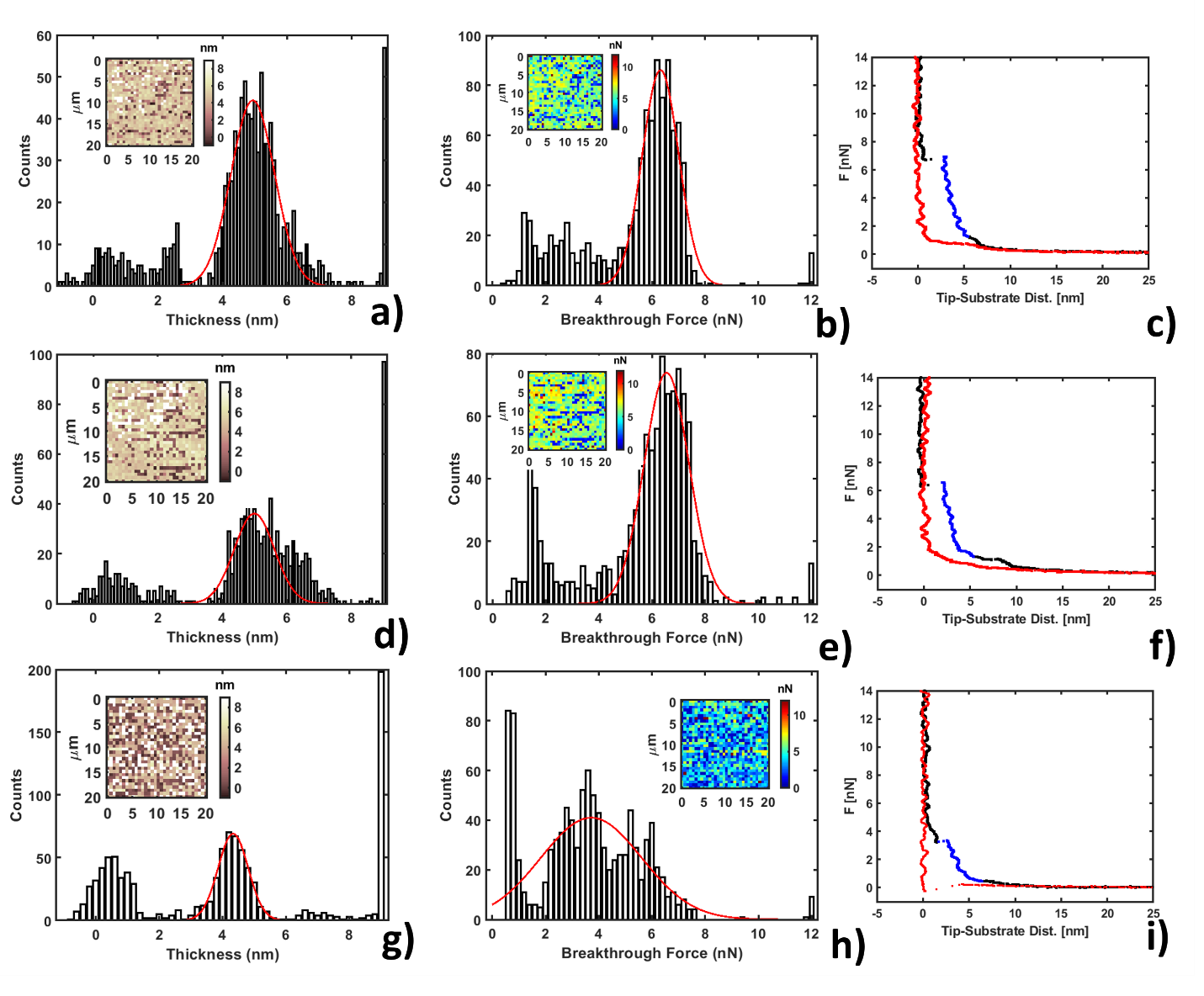


Figure SI10. Supplementary measurements of DOPC interacting with CytoTracker deep red 25 μg/ml. a) Thickness histogram of DOPC bilayer with correspondent topography image. b) Breakthrough force histogram with correspondent map; c) Example of force curve on bilayer with approaching (blue) and retracting (red). With the same order of DOPC control (a-c), DOPC after 30 min incubation with 25 μg/ml CytoTracker deep red (d-f), and after irradiating probe region with 35.4 mW/μm^2^ green laser (g-i).


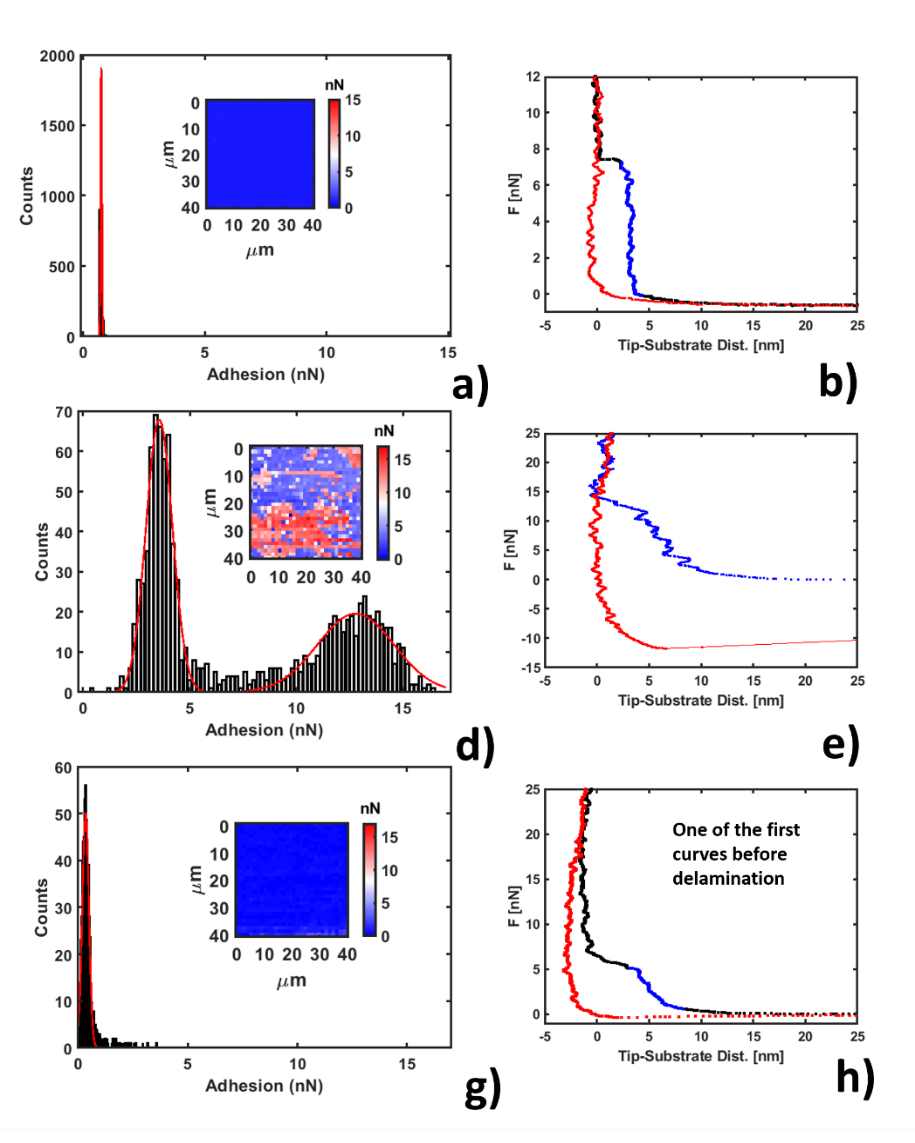


Figure SI11. Supplementary measurements of DOPC interacting with HOECHST 2.5 μg/ml. a) Adhesion force histogram of DOPC bilayer with correspondent topography image. b) Example of force curve on bilayer with approaching (blue) and retracting (red). With the same order of DOPC control (a-b), DOPC after 30 min incubation with 25 μg/ml HOECHST (d-e), and after irradiating probe region with 35.4 mW/μm^2^ blue laser (g-h).

Supporting References:

[1]https://www.olympus-lifescience.com/en/microscope-resource/primer/techniques/confocal/resolutionintro/
